# The discovery of novel heat-stable keratinases from *Meiothermus taiwanensis* WR-220 and other extremophiles

**DOI:** 10.1038/s41598-017-04723-4

**Published:** 2017-07-05

**Authors:** Wan-Ling Wu, Mei-Yi Chen, I-Fan Tu, Yu-Ching Lin, Nadendla EswarKumar, Ming-Yi Chen, Meng-Chiao Ho, Shih-Hsiung Wu

**Affiliations:** 1 Institute of Biological Chemistry, Academia Sinica, Taipei, 11529 Taiwan; 2 General Education Center, National Taipei University of Nursing and Health Sciences, Taipei, 11219 Taiwan; 3 Institute of Biochemical Sciences, College of Life Sciences, National Taiwan University, Taipei, 10617 Taiwan; 4 Department of Chemistry, National Taiwan University, Taipei, 10617 Taiwan; 5 Chemical Biology and Molecular Biophysics Program, Taiwan International Graduate Program, Academia Sinica, Taipei, 11529 Taiwan

## Abstract

Billions of tons of keratin bio-wastes are generated by poultry industry annually but discarded that result in serious environmental pollution. Keratinase is a broad spectrum protease with the unique ability to degrade keratin, providing an eco-friendly way to convert keratin wastes to valuable amino acids. In this report, a feather-degrading thermophilic bacterium, *Meiothermus taiwanensis* WR-220, was investigated due to its ability to apparently complete feather decay at 65 °C in two days. By genomics, proteomics, and biochemical approaches, the extracellular heat-stable keratinase (MtaKer) from *M. taiwanensis* WR-220 was identified. The recombinant MtaKer (rMtaKer) possesses keratinolytic activities at temperatures ranging from 25 to 75 °C and pH from 4 to 11, with a maximum keratinolytic activity at 65 °C and pH 10. The phylogenetic and structural analysis revealed that MtaKer shares low sequence identity but high structural similarity with known keratinases. Accordingly, our findings have enabled the discovery of more keratinases from other extremophiles, *Thermus* and *Deinococcus*. Proteins encoded in the extremophiles shall be evolved to be functional in the extreme conditions. Hence, our study expands the current boundary of hunting keratinases that can tolerate extreme conditions for keratin wastes biorecycle and other industrial applications.

## Introduction

Over one billion tons of animal-derived keratinous wastes, such as poultry feathers, bristles, wools, and horns, are generated annually, but lack proper applications and are therefore discarded^1, 2^. Disposal of such wastes in landfills or incinerators elevates both environmental and monetary costs. A significant obstacle in tackling these costs is the main component of these wastes, keratin, which is a protein with a highly rigid structure that resists mechanical stress and proteolysis by general proteases like pepsin, papain, or trypsin^3^. Despite the fact that keratin is regarded as the hardest-to-degrade animal protein, keratin-rich materials and poultry feather waste in particular hold great potential as a source of amino acids for animal feed and biotechnological applications^4, 5^.

In general, keratin molecules stack together to become either a coiled-coil (α-keratin; e.g., hair, nail, and wood) or β-strand stacking (β-keratin; e.g., feather) polypeptide chain^6^. The high cysteine content and consequent disulfide bridges contribute to the stability and recalcitrance, or resistance to degradation, of different keratins. In nature, a special class of proteases, named keratinase, can degrade keratin and other proteins^7^. Keratinases are commonly produced as extracellular proteases by diverse microbes, including *Bacillus* sp., other bacteria, and fungi^8^. Due to their multitude of industrial applications and the increasing biotechnological interest, keratinase and keratinolytic microorganisms have attracted wide attention. For instance, keratinase produced by *Bacillus licheniformis* has been extensively studied for its feather keratin-degrading abilities and is commercially used in the animal feed industry^5, 9^. In fact, the keratinase field has flourished owing to the studies of keratinolytic enzyme from *B. licheniformis*. Keratinases are classified in the superfamily of subtilisin-like proteases, belonging to the serine protease (S8 family)^10^. However, not all subtilisin proteases can degrade keratin, and it remains unclear which features are required for keratin degradation. Therefore, most known keratinases are identified by sequence homology with keratinase (KerA) from *B. licheniformis*. Most research in this field centers on screening novel microorganisms with high keratinase activity or using their grown medium for keratinase purification without any accompanying genetic insight^3^. Thus, the production of recombinant keratinase by heterologous expression and purification by affinity tag is relatively limited^7, 10^, as is biochemical and biophysical research into the promising proteolytic enzyme. Its ability to degrade insoluble keratin underscores the need for greater insight and improved yield in commercial or industrial applications.

In this study, we identified a thermostable keratinase (MtaKer) from the thermophile strain, *Meiothermus taiwanensis* WR-220, which grows at 55–65 °C^11^. We heterologously expressed and purified rMtaKer using the *E. coli* expression system. Our biochemical analyses revealed its suitability for industrial use, with a wide range of protease activities, especially on keratin, and high stability in extreme heat, acidic, and basic environments. Because this newly identified MtaKer shares lower sequence identity with *B. licheniformis* KerA, it serves as a new reference sequence to uncover more keratinases from extremophiles. More importantly, our crystal structure of MtaKer provides insights into substrate recognition and catalysis crucial for keratinase activity, and may serve as a blueprint for potential protein engineering.

## Results

### Keratinase ability of *M. taiwanensis* WR-220

**Figure 1:**
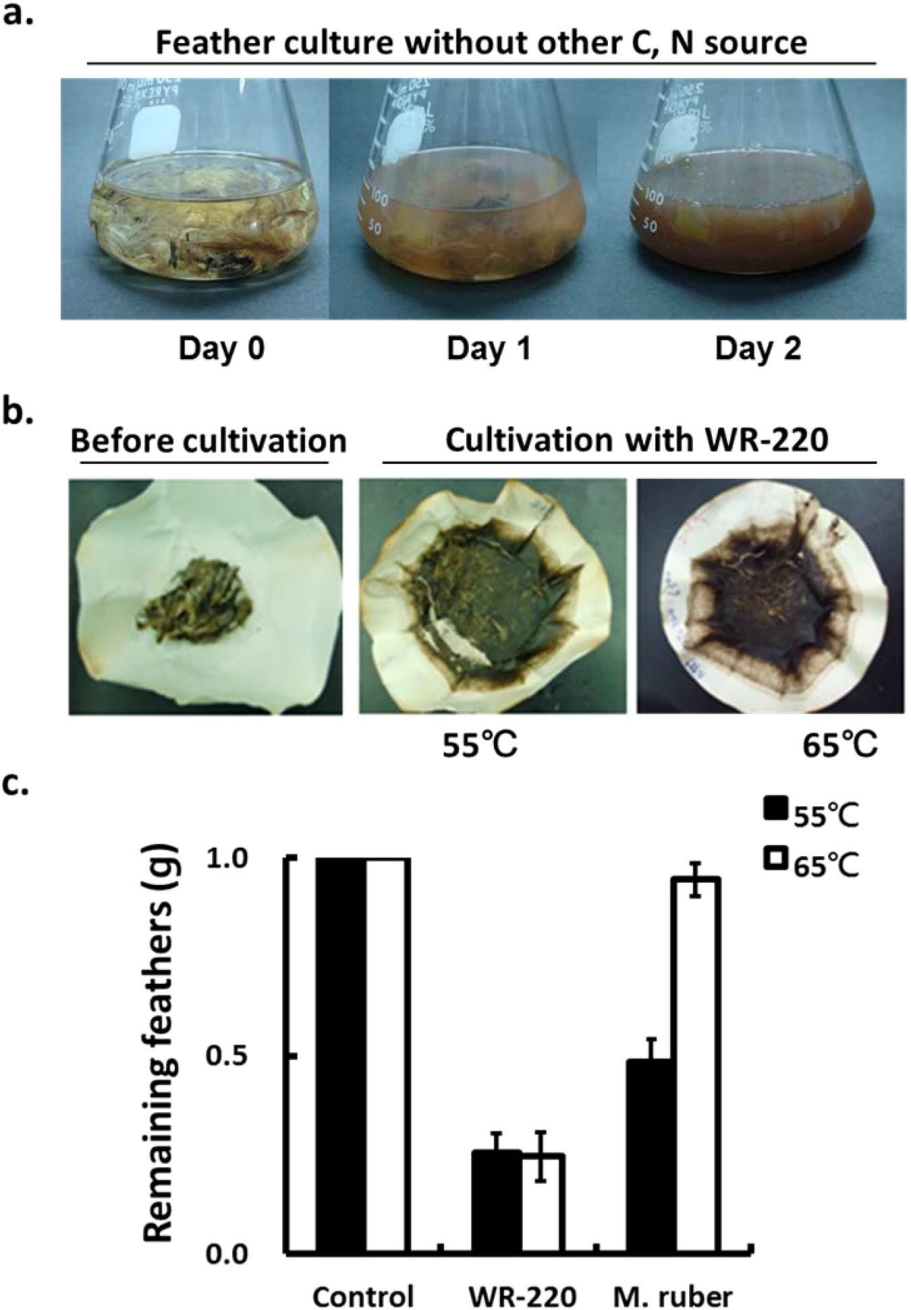
Degradation of intact chicken feathers by *M. taiwanensis* WR-220. (**a**) *M. taiwanensis* WR-220 degraded native chicken feathers at 55 °C after two days of incubation. The basal medium contained 0.5% feathers (w/v) as the sole source of carbon and nitrogen. (**b**) The remaining feathers after decay in two days culture at 55 and 65 °C were shown on filter papers. (**c**) *M. taiwanensis* WR-220 displayed high feather-degrading activity at 55 and 65 °C compared to the *Meiothermus* species, *M. ruber*. Feather-degrading capacity was determined by the weight of feathers remaining in the cultures medium containing 1% feathers (w/v) as only carbon and nitrogen source. All results were calculated from three independent experiments. Control was a non-inoculated medium.

To examine its keratinolytic ability, *M. taiwanensis* WR-220 was cultured at 55 °C in medium with insoluble feathers as the only source of nutrients. After two days of culture, the feather degradation indicated that *M. taiwanensis* produced an extracellular keratinase to degrade feather keratin as a nutrient source (Fig. 1a). *Meiothermus* species have an optimum growth temperature in a range of 55–65 °C^11^. We found that the strain WR-220 left less feather material at both 55 °C and 65 °C (Fig. 1b,c) compared to the known feather degrader *M. ruber* ^12^. Although both strains grew well on feather at 55 °C and 65 °C (Supplementary Fig. S1), only WR-220 exhibited feather degrading activity at 65 °C (Fig. 1c). These properties made WR-220 an ideal candidate for keratinase purification and identification.

### Isolation and identification of keratinase

**Figure 2:**
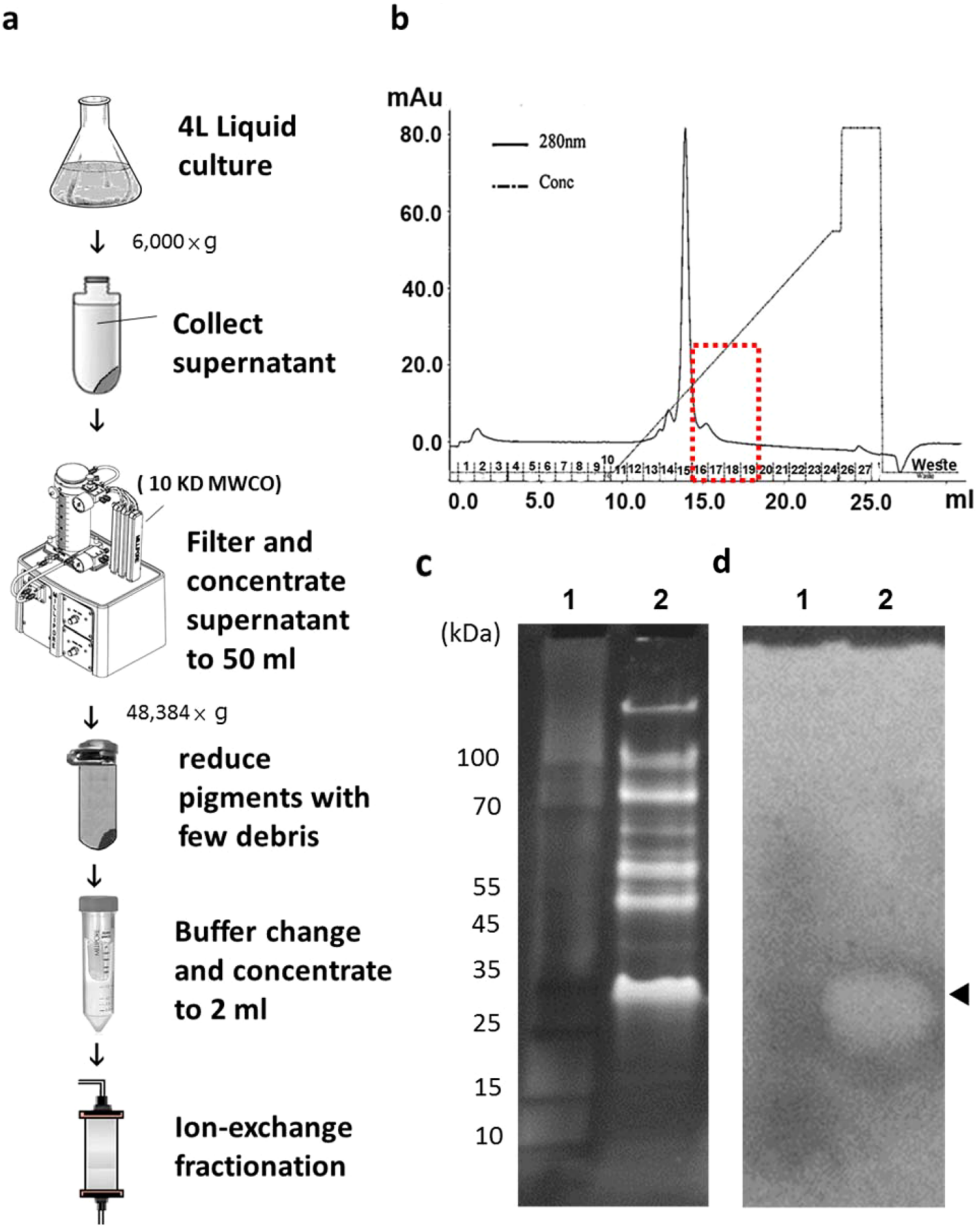
Purification of keratinase from culture medium and keratinase identification. (**a**) An overview of keratinase purification from the overnight culture medium. Each step is described specifically in Materials and Methods. (**b**) Elution profile of MtaKer after chromatography using S-Sepharose column. The proteins were eluted with a linear salt gradient. The fraction No. 15 (the major peak) is the contaminating carotenoid pigment that was eluted by 200 mM NaCl. The red box indicates the collected peak (fraction No. 16–19) containing keratinase activates. (**c**) The proteins from the collected peak were further separated by 4–20% gradient SDS-PAGE to determine the size or molecular weight of unknown proteins. Lane 1: protein markers; lane 2: the collected peak with keratinase activity. (**d**) The keratinolytic enzyme profile was evaluated by replica-agarose-zymogram analysis on the 1% agarose with keratin powder/casein as substrates. The keratinolytic activity can be visualized as a clear zone (arrowhead) and the corresponding band on SDS-PAGE was subjected to standard proteomic analysis to identify the amino acid sequence of MtaKer.

Our purification process to identify the extracellular keratinase in *M. taiwanensis* WR-220 is outlined in Fig. 2a. In brief, culture supernatants of WR-220 with the keratinolytic activity were collected after overnight culture because the extracellular keratinase could be produced constitutively under non-inducible conditions (Supplementary Fig. S2). Using tangential flow filtration with a molecular mass cut-off of 10 kDa, secreted proteins could be concentrated to very high concentrations into a small volume. The special attention should be paid to remove water-soluble pigments prior to the fractionation of extracellular extracts with ion exchange chromatography. Because a dark red intracellular carotenoid pigment produced by WR-220 has a strong absorbance at the wavelength of 280 nm, which we used to monitor the protein signal at the chromatography step^13, 14^. However, the remaining pigment still presented as a major peak in our chromatography (Fig. 2b). The collected fractions that had keratinase activity were separated by 4–20% gradient SDS-PAGE (Fig. 2c) and further identified by keratin zymography analysis on a replica agarose (Fig. 2d). Zymography clearly showed one zone containing keratinolytic activities with a molecular mass of around 30 kDa (Fig. 2c,d). The corresponding protein band with keratinolytic activity on the SDS-PAGE (Fig. 2c) was subjected to Nano-HPLC-MS/MS analysis to identify the proteins in this zone. Using our genomic sequencing result (NCBI bio project submission ID: SUB251796) as the reference database, the applied proteomics methods identified 20 proteins (see Supplemental information and Supplementary Table S1). Among them, a candidate (Mtai_v1c14470) annotated as peptidase S8/S53 subtilisin kexin sedolisin appeared to be a keratinase (Supplementary Fig. S3) despite its relatively low sequence homology with known keratinases (Supplementary Fig. S4). For example, it shared only 32.5% sequence identity with keratinase KerA from *Bacillus licheniformis* PWD-1^7, 15^. BLASTP analysis of the Mtai_v1c14470 amino acid sequence revealed greater similarity with two serine protease﻿s﻿ from the thermophilic *Thermus* (59.7% identity) and mesophilic *Deinococcus* (53.7% identity) species (Supplementary Fig. S4). However, these two proteases have never been reported for their keratinolytic activity.

### Production and determination of recombinant MtaKer keratinase

**Figure 3:**
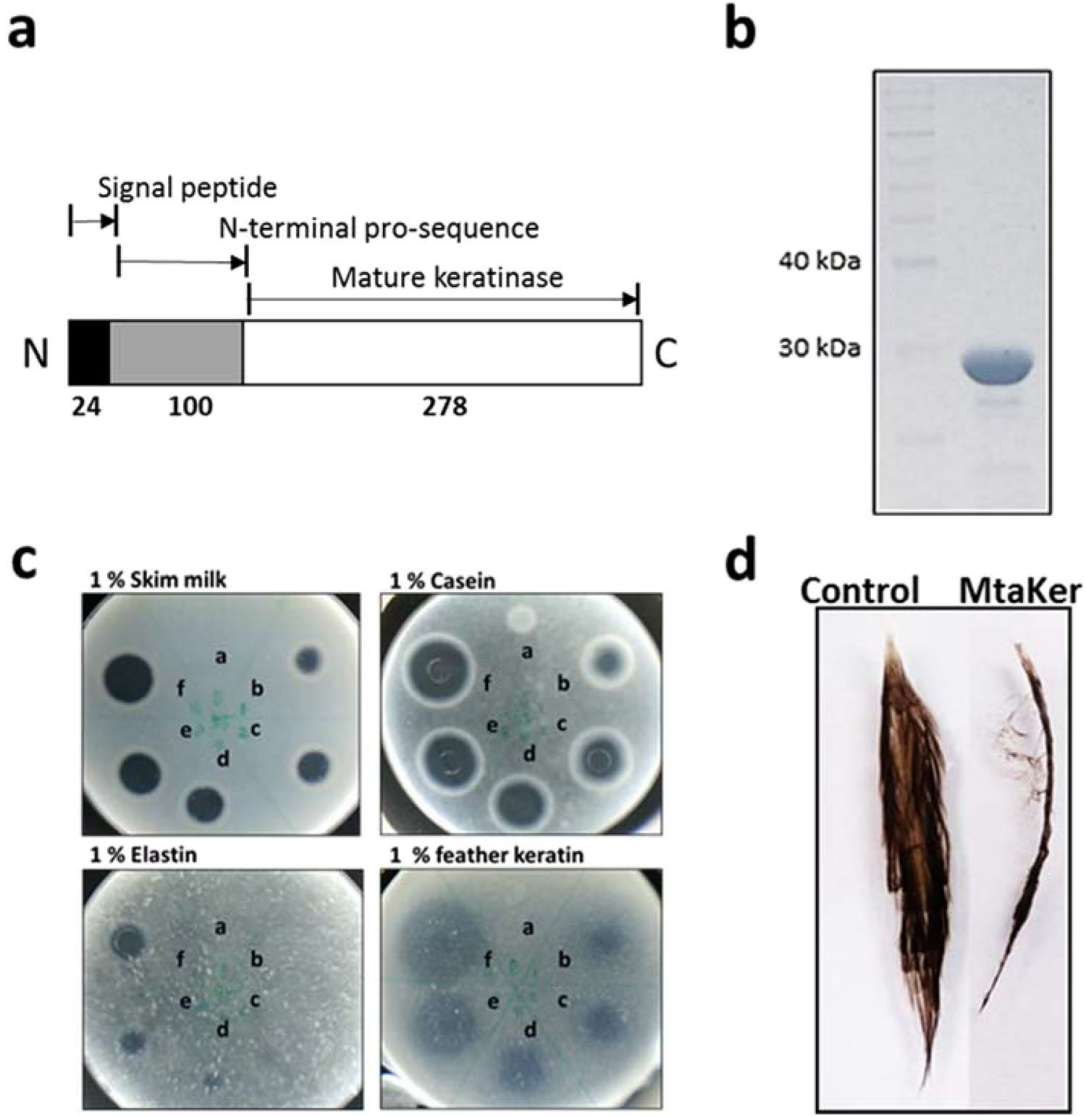
Domain structure and determination of rMtaKer keratinase proteolytic activities. (**a**) The three-domain structure of rMtaKer represents the positions of the signal peptide (pre, 24 amino acid residues), N-terminal pro-sequence (N-pro, 100 residues), and mature keratinase (278 residues). (**b**) The rMtaKer keratinase exhibited autoprocessing activity into mature keratinase with the molecular weight of 28 kDa. (**c**) A broad range of rMtaKer proteolytic activities, easily digesting 1% skim milk, casein, elastin, and feathers for 12 hours at 55 °C. For agar diffusion assay, disk filter papers impregnated with the different amount of rMtaKer were placed on the agar plate containing different protein substrates. The protease activities were assessed by the diffused digested zone. For better demonstration of protease activities, the disk papers were removed. The 0 μg, 1 μg, 2 μg, 4 μg, 8 μg and 16 μg of keratinases are labeled as a, b, c, d, and e, respectively. (**d**) The 0.1 mg of the purified rMtaKer degraded whole chicken feather after overnight incubation at 55 °C.

Like many subtilisin-like serine proteases, bioinformatic analysis indicates that Mtai_v1c14470 consists of three parts, including signal peptide, N-terminal pro-peptide (N-pro), and mature protease (Fig. 3a), and has the three highly conserved catalytic triad residues Asp39, His72, and Ser224 (Supplementary Fig. S4a)^16, 17^. To examine whether Mtai_v1c14470 (MtaKer) is the keratinolytic protease produced by WR-220, we constructed an expression plasmid carrying a signal peptide-truncated *mtaKer* gene with a C-terminal 6xhis-tag for the *E. coli* expression system. The cloned rMtaKer, including the pro-peptide and catalytic domain, has an expected molecular weight of 38.8 KDa based on amino acid sequence prediction; however, only one protein band is detected in the SDS-PAGE after purification by Ni-affinity chromatography and migrates at 28 KDa (Fig. 3b). The observed protein band at 28 KDa is consistent with the position of our zymogram activity staining (Fig. 2c). QTOF-MS analysis confirms the exact molecular weight of purified rMtaKer to be 28,468 Da, which corresponds to the molecular mass of the mature rMtaKer without the pro-peptide (Fig. 3a and Supplementary Fig. S5). We verified the proteolytic activity of the purified rMtaKer on milk, casein, elastin, and feather keratin by disk-agar diffusion assay. The results revealed that rMtaKer possesses a broad range of proteolytic activities (Fig. 3c), including powerful keratinase activity toward intact chicken feathers (Fig. 3d).

### Effects of pH and temperature on keratinase and protease activity

**Figure 4:**
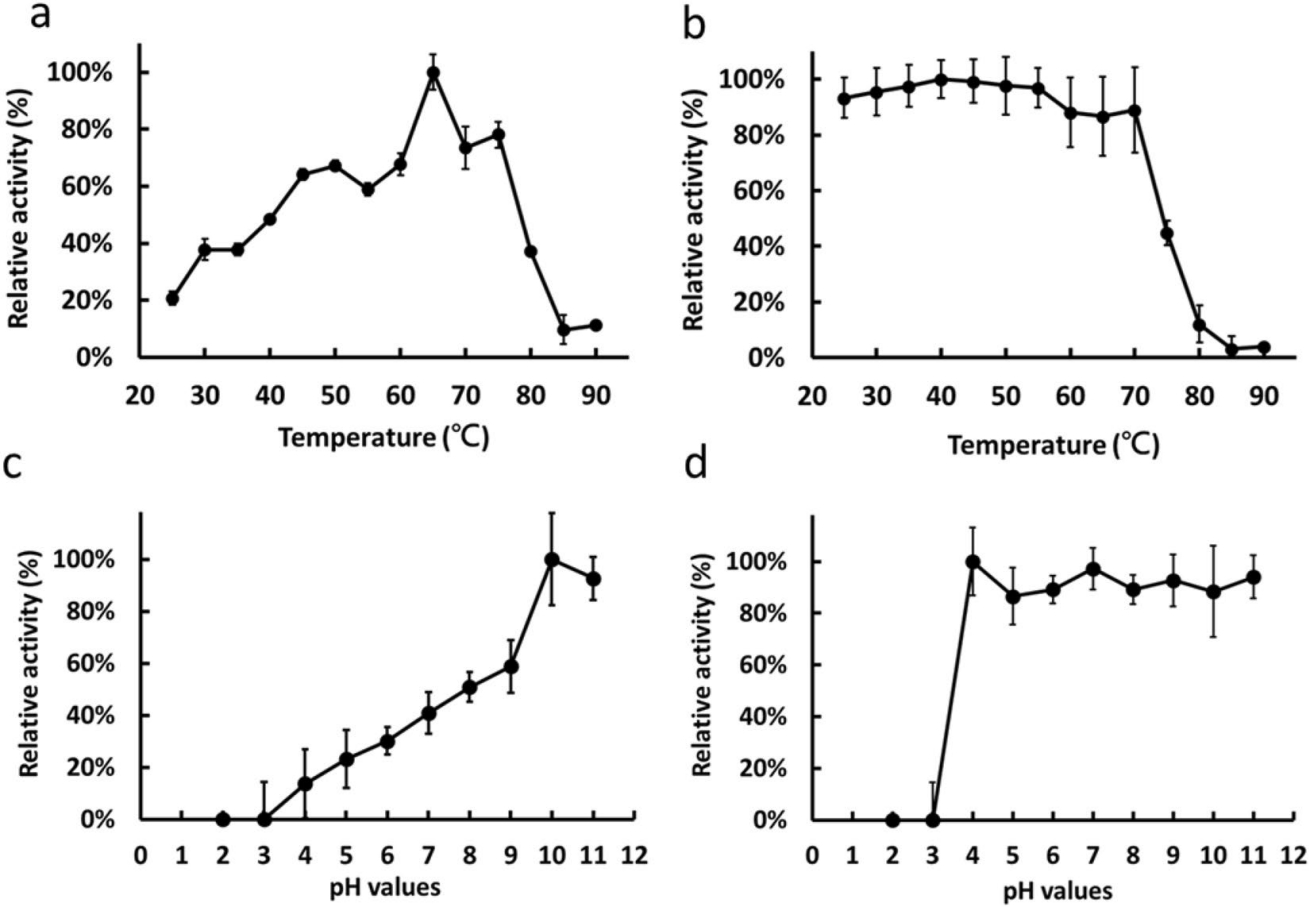
Effects of temperature and pH on the keratinolytic activity and stability of the purified rMtaKer. (**a**) The optimum temperature was detected at different temperatures 25–90 °C for 1 h, using feather keratin as substrate. (**b**) The thermal stability of rMtaKer was measured by pre-heating 5 uM of enzymes at different temperatures for 1 h and assayed under standard conditions at 55 °C. (**c**) The optimum pH of rMtaKer was determined at 55 °C in various pH buffers (pH 2~11). (**d**) The pH stability of rMtaKer was examined by pre-incubating 1 uM rMtaKer in different pH solutions at 25 °C for 1 h and assayed under standard conditions at 55 °C.

We used feather powder as a substrate to measure the enzymatic hydrolysis and quantified the free amino groups released during hydrolysis by ninhydrin assay. At the optimum temperature of 65 °C, 30 nM rMtaKer produced 1.2 mM of free amino acid from 1% (m/v) feathers at pH 8 for 1 hr. rMtaKer retained its full activity after incubating at 75 °C for 1 h (substrate is present), indicating that its keratinase activity was thermostable. However, the thermal stability profile showed that rMtaKer only retains its full keratinolytic activity after 1 h incubation (without substrate) at 70 °C and drops to half the activity level at 75 °C (Fig. 4b), demonstrating that the presence of the protein substrate increases rMtaKer thermostability. Similar phenomena have been shown in other enzymes in studies that demonstrated that heated enzymes can be stabilized by proteins when there is a net loss of surface hydrophobicity during the heating^18^. We found that the pure keratinase can retain 54% of keratinase activity with a short 5-minute incubation up to 85 °C and some protease activity with short incubation up to 95 °C (Supplementary Fig. S6a). The optimal pH profiles showed that rMtaKer is active at a broad range of pH 4–11 and reaches its maximum activity at pH 10 (Fig. 4c). As for the pH stability, pure rMtaKer maintains full activity after a 1-hour pre-incubation at pH 4 and after a 5-minute short treatment at pH 3 (Fig. 4d and Supplementary Fig. S6b).

### Crystal structure of rMtaKer

**Figure 5:**
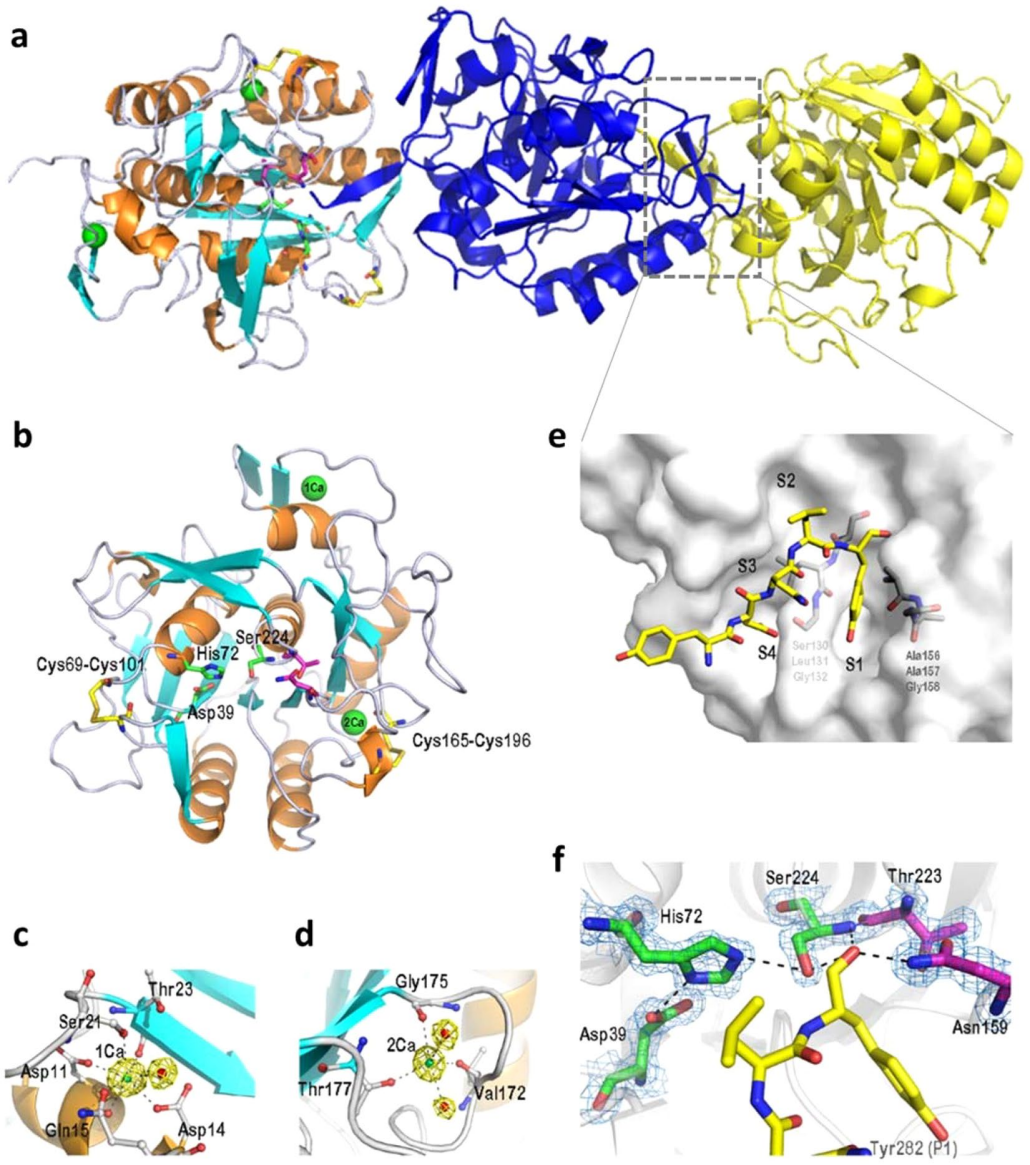
Crystal structure of rMtaKer. (**a**) Stereoview of three mature rMtaKer monomers is shown in the asymmetric unit. The trimers are named chain A (orange/cyan), chain B (blue), and chain C (yellow). (**b**) The core structure of mature rMtaKer is made of a seven-stranded parallel β sheet flanked by six α-helices (orange) and five β-sheets (cyan). The calcium ions are shown as green spheres and two intramolecular disulfide bridges are highlighted in yellow. The catalytic triad (Asp, His, and Ser) is labeled by green sticks and the oxyanion hole residues are marked by magenta sticks. (**c**) Structure of the 1Ca-binding site forms a pentagonal bipyramidal geometry with five residues and one water molecule (red ball). (**d**) Structure of the second Ca-binding site is coordinated to three residues and two water molecules. (**e**) The end part of the C-terminus (Tyr-Glu-Asn-Leu-Tyr) from C chain binds at the substrate-binding cleft (S1–S4) on the B chain of rMtaKer as shown in gray. The substrate is displayed as a yellow stick model. (**f**) Tyr282 seals the hydrophobic pocket of S1 site and several hydrogen bonds are also observed along the pocket surfaces and around the active site.

The crystal structure of mature rMtaKer was determined at a resolution of 1.50 Å and contained three monomers in an asymmetric unit (Fig. 5a). Each monomer consists of the central seven-stranded parallel β sheets flanking by six α helices and four β sheets made of two anti-parallel strands and possesses the conserved catalytic triad Asp39, His72 and Ser224 (Fig. 5b). Two calcium ions and two disulfide bridges were also found in the structure of rMtaKer. It has been reported that the calcium binding is essential for correct folding and structure stability, and the disulfide bonds can enhance the thermal stability^19, 20^. The first calcium ion (1Ca), involved in stabilizing the surface loop between α1 and β2 (Fig. 5c), is coordinated by Oδ1 of Asp11 and Asp14, carbonyl-oxygen of Asp11 and Thr23, Oγ of Ser21, Oε of Gln15, and one water molecule in a pentagonal bipyramidal manner. The second calcium (2Ca) makes contact with the main-chain carbonyl oxygen atoms of the Val172, Gly175, Thr177, and two water molecules (Fig. 5d). These two calcium binding sites are present in other members of the subtilisin superfamily, including VPN (PDB 1sh7), protease K (PDB 1ic6), and aqualysin I AquI (PDB 4dzt)^21^. We observed two intramolecular disulfide bridges in the rMtaKer structure. The first disulfide bond, Cys69-Cys101, connects the loops which are involved in substrate-binding (Fig. 5b,e). The second disulfide bond, Cys165-Cys196, is close to the 2Ca-binding site and the region carrying residues of the S1 active-site pocket (Fig. 5b,e), which was termed S1–S4 according to a previous report^22^.

Because rMtaKer is a monomer in solution, the three independent rMtaKer (chains A, B, and C) in the asymmetric unit are mostly due to the crystal packing (Fig. 5a). Intriguingly, the structural arrangement reveals an unanticipated enzyme-substrate interaction, which may also facilitate the crystal packing (Fig. 5e). Each C-terminus His-tag protrudes into the adjacent monomer as a substrate, resulting the unexpected His-tag removal at Tyr278 (Supplementary Fig. S7). The remaining residues at the C-terminal end (Tyr^278^-Glu^279^-Asn^280^-Leu^281^-Tyr^282^) of rMtaKer was buried in the active-site cavity of the adjacent subunit (Fig. 5e). Interestingly, the protruding residues Asn280 and Leu281 from the substrate formed the anti-parallel β sheet with the main chain of active-site residues 104-105. Our crystal structure clearly shows that Ser224 acts as a nucleophile to attack the carbonyl carbon of the scissile peptide bond on Tyr282 and forms a tetrahedral acyl enzyme intermediate which can be stabilized by the oxyanion hole of Asn159 and Thr223 (Fig. 5f). Meanwhile, the S1 binding pocket consisting of Ser130-Leu131-Gly132 and Ala156-Ala157-Gly158 forms hydrophobic interactions with Tyr282. Several hydrogen bonds are also formed along the pocket surfaces and around the active sites Asp39 and His72. Accordingly, the large S1 binding pocket is regarded as the major determination of substrate preference^23^.

### Discovery of keratinases from other extremophiles

**Figure 6:**
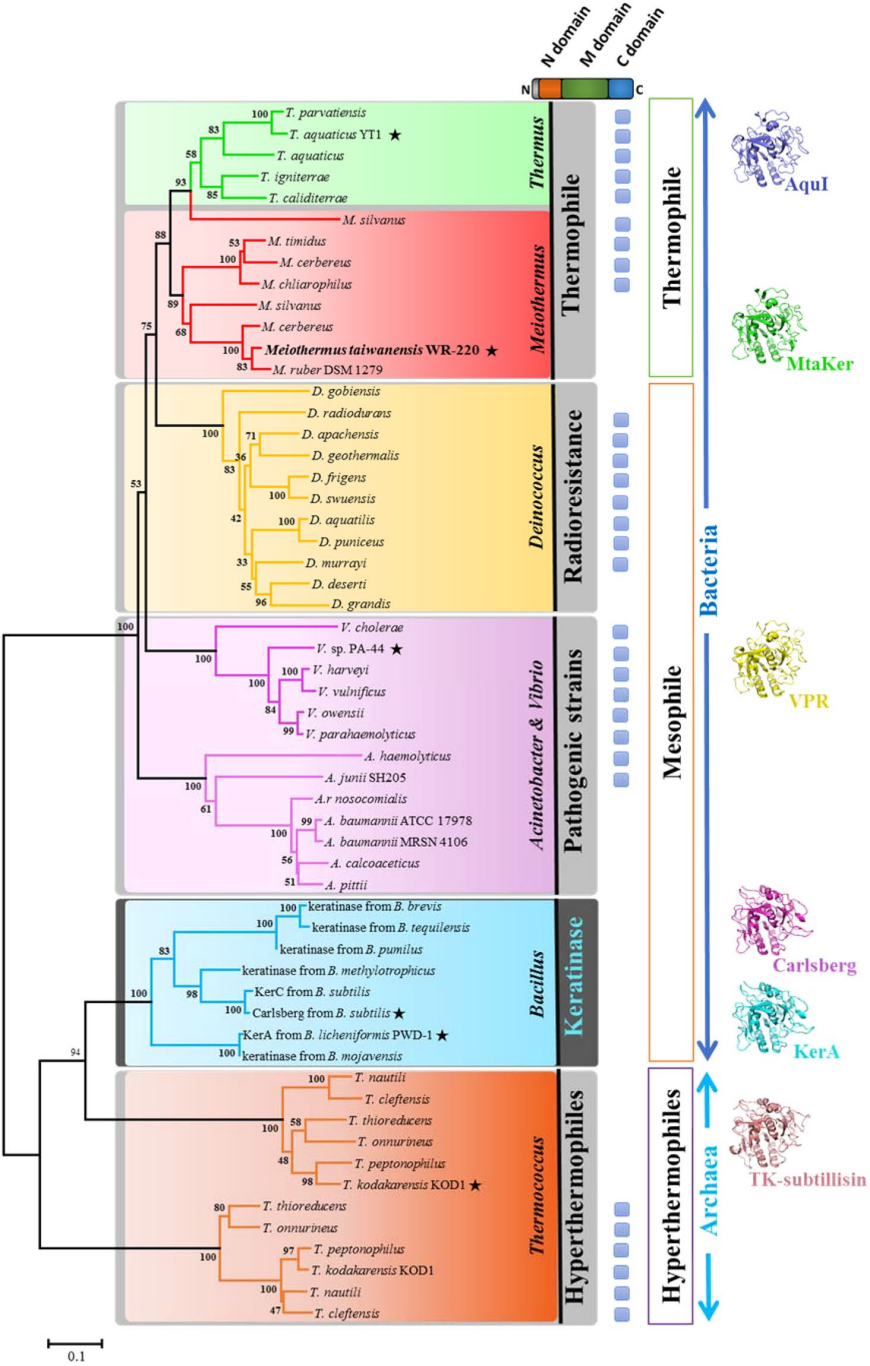
Phylogenetic tree of keratinase and the keratinolytic potential of bacterial subtilisins based on MtaKer protein sequence. The phylogenetic tree derived by the Neighbor-Joining method based on a multiple sequence alignment using MtaKer sequence. The analysis involved 57 amino acid sequences from *Thermus*, *Meiothermus*, *Deinococcus*, *Acinetobacter*, *Vibrio*, the well-known keratinase of *Bacillus* sp., and *Thermococcus*, and represented in different color squares. All sequences contain the N-terminal propeptide and mature domain, while only some possess the C-terminal propeptide (blue boxes). The asterisks indicate that protein structures are depicted on to right of the tree. The percentages of replicate trees in which the associated taxa clustered together in the bootstrap test (500 replicates) are shown next to the branches. The evolutionary distances of the tree are drawn to scale, with branch lengths in the same units as those used to infer the phylogenetic tree.

MtaKer, originally annotated as a peptidase S8/S53 subtilisin, shares approximately 30% sequence identity with well-known keratinases from *Bacillus* species, which did not immediately indicate its keratinase activity. Our assays have revealed that rMtaKer possesses thermostable keratinase activity and suggest a means to uncover other proteases with keratinase activity. To discover more keratinases, we constructed a phylogenetic tree based on the protein-sequence homology from the psychrophilic, mesophilic, and thermophilic subtilisins and well-known keratinases from *Bacillus* sp. and superimposed the available crystal structures (Fig. 6 and Supplementary Fig. S8). Indeed, MtaKer groups within the *Thermus-Deinococcus* clade, which includes *Thermus*, *Meiothermus*, and *Deinococcus* species, while the well-known keratinases instead form a separate clade and have a closer relationship with the hyperthermophilic archaeon, genera *Thermococcus*. According to the multiple alignments for the phylogenetic construction, we further found that most proteins aside from keratinase from *Bacillus* species and MtaKer carry a long C-terminal extension (Fig. 6), which is essential for the proper functions^24, 25^ (Supplementary Fig. S4a). The structural analyses showed that the reported structures of their catalytic domain from each clade (including PDB ID: 4dzt, 1sh7, 1cse, 4gi3, and 2z2x) share the same overall fold with the root-mean-square deviation (RMSD) value less than 1.8 Å. In other word, the strong structural similarity is conserved despite low sequence identity (values from 32% to 70%) (Fig. 6 and Supplementary Fig. S8). To investigate whether new keratinases could be predicted based on this information, we cloned and expressed two closely-homologous proteins, including the well-known aqualysin I (AquI) from *T. aquaticus* YT1 and an unknown peptidase (Deirad) from *D. radiodurans* R1, finding that both recombinant AquI and Deirad possess strong keratinase activity (Supplementary Figs S4a and S9). These results demonstrated that using the MtaKer protein sequence as a reference can successfully identify potential keratinases from other extremophiles that are generally annotated as peptidase or substilisin and not previously considered keratinases.

## Discussions

Protein encoded in the genus *Meiothermus* should maintain enzymatic activity at 50–65 °C, a range highly useful in industry. Strains in this genus secrete a protein complex responsible for keratin degradation, but the corresponding enzymes were previously were never reported^26, 27^. In this study, we have reported strong keratinolytic ability in the indigenous isolate *M. taiwanensis* WR-220 in culture even at 65 °C (Fig. 1), and further identified a single gene encoding the secreted keratinase MtaKer using chromatography followed by mass spectrometry (MS) proteomic analysis (Fig. 2 and Supplementary Fig. S3 and Table S1). By molecular cloning, heterologous expression in *E. coli*, and protein purification, we have confirmed that the rMtaKer possesses a broad spectrum of protease activities against insoluble substrates, including keratin (Fig. 3). The entire amino acid sequence of MtaKer is 402 residues with the expected molecular weight of 41.3 kDa (Supplementary Table S1), but we observed a protease of 28 kDa in our zymogram analysis (Fig. 2c). This molecular weight discrepancy is due to the 100 residues of the N-terminal pro-peptide that are removed to form the mature and active MtaKer by autoproteolysis, which is a known maturation process in the subtilisin family^28, 29^. A similar molecular weight discrepancy should be considered in the search for other novel keratinases.

Unlike most reported keratinases, which are only active at pH 6–9 and temperatures 25–50 °C^4, 8^, rMtaKer has keratinolytic activities at temperatures ranging from 25 to 75 °C and pH from 4 to 11, with the optimal temperature and pH at 65 °C and pH 10 (Fig. 4a,c). Moreover, rMtaKer has no loss of keratinolytic activity even after acidic treatment at pH 3 for 5 min. This suggests it advantage as an animal feed supplement, maintaining digestive function even after passing through the animal digestive systems. The purified enzyme remained active at higher temperatures, particularly in the presence of protein substrate (Supplementary Fig. S6). The high stability features of rMtaKer provide considerable advantages in heating and drying during industrial processes.

The MtaKer structure is highly similar to those of reported keratinases or subtilisin-like serine proteases (S8 family), with RMSD < 1.8 Å despite low sequence homology (Supplementary Fig. S8). Because keratinases are also serine-proteases with a broad range of protein substrate specificities, it would be expected that there are structural features similar to subtilisin-like serine proteases. The specifications for keratinase activities require further study. Interestingly, we observed that the C-terminal His-tag was removed, and the remaining C-terminal resides (Tyr^278^-Glu^279^-Asn^280^-Leu^281^-Tyr^282^) were still buried in the active site groove of the adjacent rMtaKer molecule in our crystal structure (Fig. 5e and Supplementary Fig. S7), indicating that the tag can be digested via intermolecular autoproteolysis. This is consistent with our finding that the mature rMtaKer that is purified by Ni-affinity chromatography can no longer be captured by Ni-resin after long store at 4 °C. The phenomenon may explain the unsuccessful purification of the heterologously expressed keratinases through affinity chromatography, such as the keratinolytic enzymes KerSMD, KerSMF, and fervidolysin in previous reports^30, 31^. Furthermore, the remaining end sequence of rMtaKer, residues Asn280 and Leu281, not only occupied the substrate-binding pocket but formed an anti-parallel β-sheets with main chain of residues 104–105 in the adjacent rMtaKer (Fig. 5a). The keratinase-substrate interaction extends beyond the S1 site, and the backbone of residues 104–105 is also involved in substrate interaction, which is consistent with the substrate binding mechanism of chymotrypsin-like serine proteases^32^. Our crystal structure also reveals two intramolecular disulfide bonds between Cys69-Cys101 and Cys165-Cys196. It is known that intramolecular disulfide bonds can significantly increase protein thermostability by reducing the entropy of protein unfolding states^33^. Further supporting the contribution of intramolecular disulfide bonds to the thermostability of rMtaKer, we found that breaking disulfide bonds by adding a reducing agent can largely reduce rMtaKer thermostability (Supplementary Fig. S10). Our structural comparison reveals that the Pro270 of rMtaKer is structurally conserved with Pro268 of aqualysin I. It also has been shown that Pro268 on the surface loop of thermophilic aqualysin I is important in maintaining the integrity of the overall structure at elevated temperatures^34^. This report also showed that P268T mutant significantly reduced the half-life of aqualysin I at temperatures above 90 °C and decreased the *Tm* value of 7.5 °C.

Before our work, identifying bacterial keratinases was limited to homologous sequence searches with KerA produced by *B. licheniformis* PWD-1, and there was no other useful prediction rule for keratinase amino acid sequence^8, 10^. Recent studies have focused on protein engineering to improve the catalytic efficiency, thermophilicity, anti-salt, and detergent tolerance of keratinase for industry use^30, 35, 36^. Our phylogenetic and structural analysis (Fig. 6 and Supplementary Figs S8 and S9) have uncovered more keratinases from extremophilic microorganisms, which attract notice for their potential in biotechnology and diverse industrial processes^37, 38^. Because these are in extremophiles, the extracellularly secreted keratinases shall be evolved to adapt to extreme conditions, which have great potentials in biotechnology and diverse industrial processes.

In conclusion, we identified and purified a novel heat-stable keratinase MtaKer from *M. taiwanensis* WR-220. The rMtaKer shows a powerful keratinase activity toward feather keratin, especially at high temperature 65 °C and extreme pH at 10. Our structural study further revealed that rMtaKer has striking structural resemblance of known keratinases but shares with low sequence identity. Based on the structural features combining with phylogenetic analysis, two novel keratinases were further uncovered from other extremophiles, *Thermus* and *Deinococcus*, that expands the boundary of hunting more useful keratinases. Therefore, our results revealed that not only rMtaKer has potential as a less expensive and environmentally-friendly technology to degrade keratinous wastes, but also our methodology and sequence data provide new insight into the identification or design of highly efficient keratinases.

## Methods

### Bacterial culture and source of keratinase gene

The strains *M. taiwanensis* WR-220 (ATCC BAA-400) isolated from Wu-rai Hot Spring in northern Taiwan and *M. ruber* DSM 1279 ^T^ were grown under aerobic conditions at 55 °C in *Thermus* modified (TM) medium^11, 39^. *M. ruber, T. aquaticus* YT1, and *D, radiodurans* R1 were purchased from Bioresource Collection and Research Center, Taiwan, and were cultured in TM medium. The genes encoding MtaKer, AquI, and Deirad, were PCR amplified from the genomic DNA of *M. taiwanensis* WR-220, *T. aquaticus* YT1, and *D, radiodurans* R1, respectively.

*E. coli* DH5α and BL21 (DE3) competent cells (Novagen, Madison, WI) were used as hosts for genetic manipulations of plasmids and for the overexpression of proteins, respectively. *E. coli* strains were grown in Luria-Bertani (LB) medium at 37 °C. Kanamycin (30 g/ml) and/or ampicillin (100 g/ml) was added for plasmid selection.

### Feather degradation by *M. taiwanensis* WR-220 and M. ruber DSM1279

For intact chicken feather degradation, *M. taiwanesis* WR-220 and *M. ruber* DSM1279 were aerobically cultured in 50 ml of a carbon- and nitrogen-deprived medium containing an appropriate amount of chicken feathers as the nutrients. The chicken feathers were rinsed with water and air-dried, then added to the culture medium and autoclaved^12^.

### Isolation of MtaKer from *M. taiwanensis* WR-220 culture medium

*M. taiwanensis* was cultured in 4 L of TM medium under non-inducible conditions after overnight incubation at 55 °C. The culture medium was centrifuged to remove microbes and insoluble residues, and then filtered through a 0.22 μm filter. The cell-free supernatant was concentrated and buffer exchanged to 50 mL PBS buffer (pH 7.4) by a tangential flow filtration process using a Labscale™ TFF system (Millipore, USA) with three Biomax-10 Pellicon XL ultrafiltration cassettes. The lipophilic carotenoid pigments are embedded in the lipid portion of cells or cell debris and can largely be removed by centrifuged for 1 h at 4 °C at 20,000 rpm (48,384 × *g*) to reduce the interference during extracellular keratinase extraction without adding any organic solvents. The resulting clear solution was further concentrated to 2 mL using Amicon-Ultra 15 centrifugal filter devices. The concentrated sample was subjected to cation exchange chromatography on a 1 mL Resource S-column (GE Healthcare, USA), pre-equilibrated with 50 mM sodium acetate buffer (pH 5.0). The protein was eluted with a linear gradient of NaCl from 0 to 1 M in 50 mM sodium acetate. The absorbance was monitored and recorded at 280 nm. 10 μl of resulting fraction was further analyzed by SDS-PAGE using 4–20% gradient minigels according to the Laemmli method^40^. The keratinolytic activity was determined by keratin zymography.

### Keratin Zymography assay

After electrophoresis, the SDS-PAGE was washed twice in 1.5% Triton X-100 solution. This step allows proteins to refold in PAGE and is essential for keratinases to retain their activities for replica-agarose-zymography assay. Before Sypro Ruby staining, the washed PAGE was then replica-plated onto the 1% agarose plate containing feather powder/casein in PBS buffer. Casein promotes the rapid and efficient detection of protease activities, while feather powder is used to measure feather/keratin degrading ability. The keratinase activity was detected by clear zone appearing on the replica-gel. For better visualization of clear zone, the replica-agarose was stained by Coomassie Brilliant Blue R250, followed by de-staining with a solution containing 40% methanol and 7% acetic acid. After locating the keratinase on the replica-agarose, the corresponding protein band on PAGE was subject to standard proteomic analysis (details in the supporting “Materials and Methods” section) to identify the amino acid sequence of MtaKer for recombinant MtaKer production.

### Construction of keratinase expression plasmid

The keratinase genes (*mtaker*, *aquI*, and *deirad*) without the signal sequence were PCR amplified using Phusion Flash High-Fidelity PCR Master Mix (Thermo Scientific, USA) and the primers listed in Supplementary Table S2. The amplified fragments were cloned into pET9 or pET28 expression vector (Novagen) containing the C-terminal His-tag. The plasmids encoding keratinase were extracted from the successful transformants for sequencing (Genomics BioSci & Tech, Taipei, Taiwan) and expression of recombinant keratinase in *E. coli*.

### Purification and preparation of rMtaKer

The successful expression construct was transformed into *E. coli* (DE3) ArticExpress. *E. coli* was cultured overnight at 37 °C in 20 mL of LB medium containing 50 µg/mL of kanamycin. The cultures were transferred into 1 L of fresh medium and induced with 0.4 mM isopropyl-β-D-thiogalactopyranoside at *A*_600_ = 0.6–0.8, followed by overnight expression at 20 °C. The cells were harvested by centrifugation at 6,000 × *g* for 30 min, resuspended in cold lysis buffer (20 mM imidazole, 250 mM NaCl, and 50 mM HEPES, pH 8.0), and disrupted by French press. The *E. coli* debris was removed by centrifugation at 8,000 × *g* for 30 min. The recombinant keratinases were purified by Ni-affinity chromatography (Novagen) and eluted with an increasing imidazole gradient. The eluted keratinase was concentrated and buffer-exchanged using Amicon Ultra filters with a 10-kDa cutoff (Millipore) into keratinase buffer (10 mM CaCl_2_, 150 mM NaCl, and 50 mM HEPES, pH8.0). The molecular weight of purified rMtaKer was determined by protein electrophoresis and mass spectrometry. The enzyme was protected by United States Patent 9,434,934^41^.

### Crystallization, data collection, and structure determination

The keratinase was crystallized in 0.2 M lithium sulfate, 0.1 M sodium acetate, and 50% PEG400 at 19 °C using the sitting drop vapor diffusion method. Crystals were flash-cooled in liquid nitrogen prior to data collection. X-ray diffraction data were collected at 15 A beamline of National Synchrotron Radiation Resource Center on an ADSC Q315 detector at 100 K. Data were processed using the HKL2000 program suite^42^. Data processing and refinement statistics are summarized in Supplementary Table S3. The structure of the keratinase (PDB ID: 5WSL) was determined by molecular replacement using the crystal structure of Aqualysin I (PDB ID: 4DZT). Models were iteratively rebuilt in COOT and refined in Refmac5^43, 44^.

### Paper disk-agar diffusion assay for keratinase and protease activity

Protease or keratinase activities were examined using 1% agarose plates containing 1% skim milk, casein, elastin, or feather powder in keratinase buffer. Sterile filter paper disks of 5 mm diameter were impregnated with different concentrations of rMtaKer (20 μl/disk) and lightly placed on the surface of the agar for 12 hours of incubation at 55 °C. The proteolytic activities were determined by the appearance of a clear zone after removed each filter paper disks from agar plates.

### Assay of keratinase activity

The chicken feathers that were rinsed, air-dried and ground by ball mill were used as the substrate for our keratinase activity. The reaction mixture contained enzyme and 1% non-sterile feather powder in a total volume of 0.2 ml keratinase buffer. The reaction was carried out in PCR tubes at different temperatures for one hour in a shaking incubator. Boiling at 100 °C for 3 min terminated the reactions, and the free amino acids released from the degraded feather powder were quantified by the ninhydrin method^45, 46^ using glycine as a standard. The mixture was centrifuged at 6,000 × *g* for 1 min to remove the insoluble substrates. The 50 μl supernatant diluted with 50 μl keratinase buffer was mixed with 50 μl ninhydrin reagent (Sigma-Aldrich) and boiled for 10 min at 100 °C before centrifugation for cooling at room temperature. 75 μl of supernatant was added to 125 μl of EtOH for a total volume of 0.2 ml in a 96-well microtiter plate for an ELISA reader (MRX Revelation, Dynex Technologies) at 570 nm.

### Determination of temperature/pH optimum and stability

The optimal reaction temperature of rMtaKer was investigated in the temperature range 25–95 °C in 150 mM NaCl, 10 mM CaCl_2_, 50 mM HEPES at pH 8.0, while the optimal pH experiment was carried out over a pH range of 2–11 at 55 °C, using 1% feather powder as a substrate. The control reaction contained buffer and feather powder only. To determine the optimal pH, enzymatic reactions were in the same buffer system at different pH and then neutralized to pH 8.0 for the ninhydrin assay. The pH stability was measured by pre-incubating 1 uM rMtaKer in different pH solutions at 25 °C for 1 h, and then neutralizing to pH 8.0 for the standard procedure detecting keratinase activity at 55 °C. A buffer (400 mM Citric acid, 400 mM NaH_2_PO_4_, 500 mM NaCl, 4 mM CaCl_2_) and B buffer (400 mM CAPS, 400 mM TAPS, 500 mM NaCl, 4 mM CaCl_2_) were prepared prior to measuring the effect of pH on enzymatic activity. The different pH solutions were mixed with equal volumes of 1.25 ml A and B buffer and then adjusted to the desired pH value with NaOH or HCl to a final volume of 10 ml with ddH_2_O. It is worth mentioning that the overall enzyme activity in the mixed buffer system is less than in the HEPES buffer, suggesting the mixed buffer system is not ideal for enzyme activity. However, the mixed buffer was used to maintain the ionic strength and chemical compound levels across the pH range. The thermal stability was measured by pre-heating 5 uM of enzymes at various temperatures for 1 h before assaying under standard conditions at 55 °C. Meanwhile, the remaining protease activities were examined by the paper disk-agar diffusion assay using the 1% skim milk agar plate. The non-heated enzyme was considered as a control (100%).

### Phylogenetic analysis of keratinase protein sequence

The phylogenetic analysis was constructed in MEGA6^47^ using the Neighbor-Joining method^48^. The percentage of replicate trees in which the associated taxa clustered together in the bootstrap test (500 replicates) is shown next to each branch^49^. The evolutionary distances were calculated using the Poisson-correction method at the amino acid level and are in the units of the number of amino acid substitutions per site. The analysis involved 57 amino acid sequences with their accession number obtained from GenBank (Supplementary Table S4). All positions containing gaps and missing data were eliminated. There were a total of 285 positions in the final dataset.

## Electronic supplementary material

Supplemental information
