# Supplementary material for: The discovery of novel heat-stable keratinases from Meiothermus taiwanensis WR-220 and other extremophiles
Source: Sci Rep. 2017 Jul 5;7:4658. doi: 10.1038/s41598-017-04723-4 (PMC5498600; doi:10.1038/s41598-017-04723-4)
Supplement: Supplementary file 1 — Supplemental information [file 41598_2017_4723_MOESM1_ESM.pdf]

Supplemental information

The discovery of novel heat-stable keratinases from *Meiothermus taiwanensis* WR-220 and other extremophiles

Wan-Ling Wu[a], Mei-Yi Chen[a] , I-Fan Tu[a], Yu-Ching Lin[a], Nadendla EswarKumar[a], Ming-Yi Chen[b], Meng-Chiao Ho[a,c*], and Shih-Hsiung Wu[a,c,d,e*]

[a] Institute of Biological Chemistry, Academia Sinica. Taipei 11529, Taiwan

[b] General Education Center, National Taipei University of Nursing and Health Sciences, Taipei 11219, Taiwan.

[c] Institute of Biochemical Sciences, College of Life Sciences, National Taiwan University, Taipei 10617, Taiwan

[d] Department of Chemistry, National Taiwan University, Taipei 10617, Taiwan

[e] Chemical Biology and Molecular Biophysics Program, Taiwan International Graduate Program, Academia Sinica, Taipei 11529, Taiwan

*Corresponding authors.

Supporting Materials and Methods

Feather degradation assay

For feather degradation, 1% (*v/v*) inoculum of the overnight culture was added to 50 ml of a carbon- and nitrogen-deprived medium supplemented with 0.5-3% (*w/v*) intact chicken feathers and Castenholtz salts solution at 55°C and 65°C for 2–4 days. The remaining feathers after decay during the cultivation were analyzed by filtering, drying, and weighing from three independent experiments. The number of viable cells in the culture was determined by colony-forming unit (CFU) assay, plating serial dilutions of culture and counting the colonies formed on the TM agar. The amount of amino acids released from the decayed feathers was quantified by the colorimetric ninhydrin method[1].

The intact rMtaKer molecular weight determination.

Purified rMtaKer was diluted with 50% methanol and 1% formic acid. Five pmols of rMtaKer was injected via a PicoTip (New Objective, Cambridge, MA) with a syringe pump (Harvard Apparatus, MA) and held at a flow rate of 1 μl/min throughout the analysis. The mass of intact proteins was determined using Waters Synapt G2 HDMS mass spectrometer (Waters, Milford, MA). The acquired spectra were deconvoluted to single-charge state using MaxEnt1 algorithm of the MassLynx 4.1 software (Waters).

Keratinase identification by mass spectrometry analysis

A single protein band with keratinase activity was excised for in-gel digestion with trypsin. The tryptic peptides were desalted for LTQ-Orbitrap XL hybrid mass spectrometer analysis (Thermo Electron, Bremen, Germany) as previously described[2]. Mass spectra were processed using MaxQuant software, version 1.5.1.2[3]. The derived peak lists were searched using the built-in Andromeda search engine[4] against a protein database constructed from *M. taiwanensis* WR-220 genomic sequence as the reference (NCBI bioproject submission ID: SUB251796 and bioproject ID: PRJNA205607). All the identified peptides and proteins from this proteomics analysis are provided in Supplementary Table S1.

The supplementary results:

Table S1. Results of the MS/MS ion search for the digested protein bands excised from the gel and analyzed by LTQ Orbitrap MS.

No.	WR-220 protein ID	Protein description	Mass (Da)	Score [a]	Unique peptides [b]
1	Mtai_v1c00500	Cys/Met metabolism pyridoxal-phosphate-dependent protein	39274.10	105.65	_AQENAQELALR_
2	Mtai_v1c01410	Histone family protein DNA-binding protein	10613.50	78.964	_VQLTGFGTFEVR_
3	Mtai_v1c06600	CheA signal transduction histidine kinase	99410.94	74.415	_ILEAAPR_
4	Mtai_v1c07900	Dipeptide ABC transporter substrate-binding protein	69943.04	97.904<br>129.76<br>149.74<br>87.216<br>66.939<br>74.611	_AQEIWADELPALPLR_<br>_NLNTGAVQIPTK_<br>_TANAPSSVVFADDFIQR_<br>_TQQLFIEQWR_<br>_VLGGDFLNVISNQSIK_<br>_WNANSSIELVR_
5	Mtai_v1c11780	Translation elongation factor Ts	22179.57	73.499	_EQTGAGM(ox)SDVK_
6	Mtai_v1c11980 /*holB*	DNA polymerase III, delta subunit	32791.81	42.396	_IILIAPSR_
7	Mtai_v1c14270	Dihydrolipoamide dehydrogenase	48669.89	163.45<br>113.95<br>61.815<br>106.6<br>41.876<br>113.73<br>89.466<br>133.89	_ANTGGVEYLFK_<br>_ASHAHPTLAEVLK_<br>_EQALGGTCLR_<br>_GVVEYEGGEPLVADR_<br>_ILGVHIIGPR_<br>_IPNTDGLGLENVGLSTDER_<br>_VEGPEGVQELETER_<br>_YLGHGTILTPNK_
8	Mtai_v1c14470 (MtaKer)	Peptidase S8 and S53 subtilisin kexin sedolisin	41364.34	136.96<br>276.38<br>138.42<br>165.63<br>115.77<br>224.03<br>140.11	_DACQFSPAR_<br>_NAIVGNATSGVVSNAGR_<br>_RSPNLLLYSNY_<br>_SPNLLLYSNY_<br>_TLPLSGTFTYSNTGSGVNAYIIDTGIR_<br>_VLNCSGSGSNSGVIAGVDWVR_<br>_VSHSEFGGR_

				251.05	_VTAGITVGATTSTDAR_
9	Mtai_v1c15280	Extracellular solute-binding protein family 5	54760.87	62.088	_LSGLPVIPDGILR_
				66.606	_VANLLSGAVSIITPVAAK_
10	Mtai_v1c16010	Aldehyde dehydrogenase	58432.44	87.083	_GAVLM(ox)NLAEVLTR_
11	Mtai_v1c16410	4-hydroxyphenylacetate degradation bifunctional isomerase/decarboxylase, HpaG2 subunit	29631.04	82.578	_FM(ox)TLEPGDVLLTGTPK_
12	Mtai_v1c16740	IclR family transcriptional regulator	77375.64	57.102	_LAAFLGK_
13	Mtai_v1c17800	Extracellular solute-binding protein family 5	64427.66	94.688	_VEYRPIDFNELVR_
14	Mtai_v1c21360	Phosphonate ABC transporter, periplasmic phosphonate-binding protein	32180.94	148.52	_GIAAALQGIK_
				117.25	_HDAAVLAVLNK_
				140.77	_IDYM(ox)IPAQDSDYDVVR_
				110.34	_LLFNLYR_
15	Mtai_v1c22570	Extracellular ligand-binding receptor	41060.14	57.414	_AQRPDLVYYGGIYDK_
16	Mtai_v1c22650	Extracellular solute-binding protein family 5	57736.95	123.76	_ASDAPISPGIFGYTK_
				162.97	_GQFVELVR_
				71.268	_IGSYEYNPNLAR_
				123.72	_IVPLLVER_
				101.75	_LISPELASSFAFLLR_
				69.379	_LSARPEIEVVTTPSVR_
				83.226	_M(ox)ALVETGQAHVAVR_
				106.29	_QLLAQAGYNAQNPLR_
				159.34	_TIFFYFNQAK_
				105.2	_TLTLFLR_
				102.05	_VAEAVQSQLR_
				92.856	_VSAFEVVDSHTLR_
				98.105	_FHDGTDFNAEAVK_
17	Mtai_v1c23660 /*fabZ*	beta-hydroxyacyl-(acyl-carrier-protein) dehydratase FabZ	16304.07	208.63	_AEATLSFVLR_
				58.864	_FKKPVVPGDTLILEGELLAYR_
				87.419	_KPVVPGDTLILEGELLAYR_
				131.06	_MDIYEILK_
				96.668	_M(ox)DIYEILK_
				130.22	_YPFLLIDR_
18	Mtai_v1c25240	Peptidase M20	39508.72	101.97	_GTTLGPNVIK_

				99.732<br>83.869	_GVNAIVELAHQVLR_<br>_VEALQDWDK_
19	Mtai_v1c26580	Nucleoside-diphosphate kinase	15332.74	111.61	_GLTGEIINR_
20	Mtai_v1c27560	Riboflavin synthase, alpha subunit	22489.88	143.55<br>56.551	_GFAVELAQETLR_<br>_LGGHLVTGHVDGR_

[a] The threshold of peptide identification score is 40 and calculated according to the standard MaxQuant.

[b] The unique peptide sequences are determined by MaxQuant.

Table S2. Primers used in this study.

Gene	Primer sequence (5’ to 3’)
mtaker	**F:** 5’ TTAAGAAGGAGATATACCATGCTAGCCCCGGTGCTAGGA
	**R:** 5’ GATTGGAAGTAGAGGTTCTCTGCGTAATTGCTGTACAGCAGCAGGTTG
aquI	**F:** 5’ GGGAATTCCATATGGTTTTGGGTGGTTGTCAGATGGCC
	**R:** 5’ CCCAAGCTTACCACTCCCCGAGGAGAGC
deirad	**F:** 5’ AATTCATATGAGCACCCCGGCGGCCAG
	**R:** 5’ ATTTAAGCTTCGCGCCGCTGCCGGTGTAGAGC

Table S3. The statistics table for the MtaKer X-ray crystal structure

**Data Collection**	5WSL
Space group	$P2_1$
Unit-cell parameters	
a, b, c (Å)	59.2, 67.1, 92.9
α, β, γ (°)	90, 96.1, 90
Resolution (Å)	30.00 – 1.50 (1.53 – 1.50)*
No. of measured reflections	112414 (5502)
Completeness (%)	97.3 (95.6)
Redundancy	3.9 (3.8)
Mean I/σ (I)	10.5 (2.6)
R_{means} (%)	12.4 (95.4)
**Structure Refinement**	
No. of reflections	106459
R_{work} (%)	14.5
R_{free} (%)	17.5
R.M.S.D. bond lengths (Å)	0.0221
R.M.S.D. bond angles (°)	2.00
No. of atoms	
Protein	2,080
Ligand	51
Water	660
Average B factor ($Å^2$)	
Protein	12.4
Ligand	20.1
Water	26.0
Ramachandran plot (%)	
Most favored region	98.3
Allowed region	1.7
Outliners	0

* Highest resolution shell is shown in parenthesis

Table S4. The information of amino acid sequences used in phylogenetic analysis of the subtilisin family (corresponding to Figure 6).

	Genus	Species	Accession	a.a	N/M/C domain[b]
Thermophile	*Thermus*	*T. parvatiensis*	gi:740193282	513	N/M/C
		T. aquaticus YT1	gi:927055382	513	N/M/C
		T. oshimai	gi:517273000	508	N/M/C
		T. igniterrae	gi:648483162	509	N/M/C
		T. calidíterrae	gi:740203210	516	N/M/C
	Meiothermus	*M. silvanus*	gi:753938741	516	N/M/C
		M. timidus	gi:648543034	511	N/M/C
		M. chliarophilus	gi:654420930	511	N/M/C
		M. cerbereus	gi:654400432	511	N/M/C
		M. silvanus	gi:502923274	403	N/M
		M. cerbereus	gi:654402426	403	N/M
		M. taiwanensis WR-220	This study	402	N/M
		M. ruber DSM 1279	gi:481063113	395	N/M
**Mesophile**	*Deinococcus*	*D. gobiensis*	gi:504496884	412	N/M
		D. radiodurans	gi:653293282	532	N/M/C
		D. aquatilis	gi:517840827	524	N/M/C
		D. puniceus	gi:1028460050	527	N/M/C
		D. deserti	gi:502015806	402	N/M
		D. grandis	gi:972321863	397	N/M
		D. murrayi	gi:653256904	521	N/M/C
		D. apachensis	gi:518416618	518	N/M/C
		D. geothermalis	gi:499849381	514	N/M/C
		D. frigens	gi:657676833	528	N/M/C
		D. swuensis	gi:746728693	536	N/M/C
	Vibrio	*V. cholerae*	gi:648216640	526	N/M/C
		V. sp. PA-44	gi:27464257	530	N/M/C
		V. harveyi	gi:756152385	530	N/M/C
		V. vulnificus	gi:782703901	530	N/M/C
		V. owensii	gi:780123500	534	N/M/C

		V. parahaemolyticus	gi:927313399	534	N/M/C
	Acinetobacter	*A. haemolyticus*	gi:490777814	533	N/M/C
		A. junii SH205	gi:262313093	526	N/M/C
		A. calcoaceticus	gi:973453708	392	N/M
		A. pittii	gi:748190990	393	N/M
		A. nosocomialis	gi:549997318	392	N/M
		A. baumannii ATCC 17978	gi:193077548	395	N/M
		A. baumannii MRSN 4106	gi:831394179	374	N/M
	Bacillus[a]	*B. brevis*	gi:514482320	383	N/M
		B. tequilensis	gi:846451730	383	N/M
		B. pumilus	gi:300429856	383	N/M
		B. methylotrophicus	gi:443298521	379	N/M
		KerC *from B. subtilis*	gi:164664938	381	N/M
		Carlsberg *from B. subtilis*	gi:727929352	362	N/M
		KerA *from B. licheniformis* PWD-1	gi:998767	379	N/M
		B. mojavensis	gi:50363121	379	N/M
**Hyperthermophiles**	*Thermococcus*	*T. nautili*	gi:757137176	426	N/M
		T. cleftensis	gi:851288153	419	N/M
		T. thioreducens	gi:943598788	422	N/M
		T. onnurineus	gi:501568435	426	N/M
		T. peptonophilus	gi:1011495002	424	N/M
		T. kodakarensis	gi:499569843	422	N/M
		T. thioreducens	gi:943599890	657	N/M/C
		T. onnurineus	gi:501567881	656	N/M/C
		T. peptonophilus	gi:1011494982	662	N/M/C
		T. kodakarensis	gi:57159948	663	N/M/C
		T. nautili	gi:757138200	666	N/M/C
		T. cleftensis	gi:504602079	658	N/M/C

[a] The well-known keratinase from *Bacillus* sp.

[b] All sequences contain N-terminal propeptide (N) and mature (M) domain, while some of them possess C-terminal propeptide (C).

Figure S1.

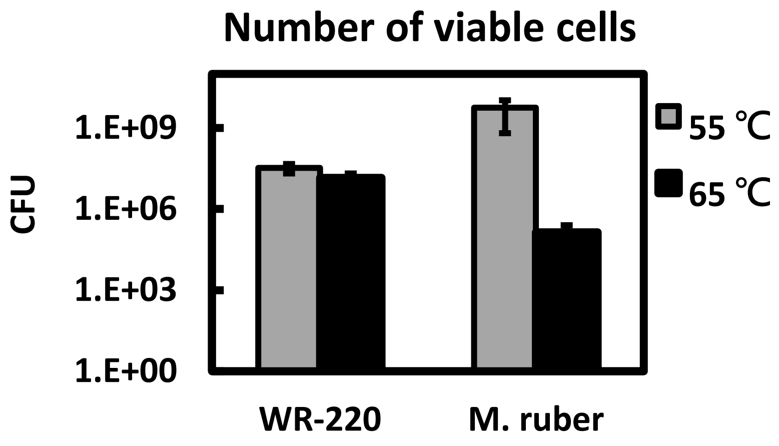

Fig. S1. Number of viable cells in culture during degradation of intact chicken feather. Cultures of WR-220 or *M. ruber* were cultivated in medium containing 2% feathers (1g/50 ml) as only carbon and nitrogen source at 55 and 65°C for two days. The number of viable colony-forming unit (CFU) per ml for each strain was counted by dilution plating. The present data are from at least three independent experiments.

Figure S2.

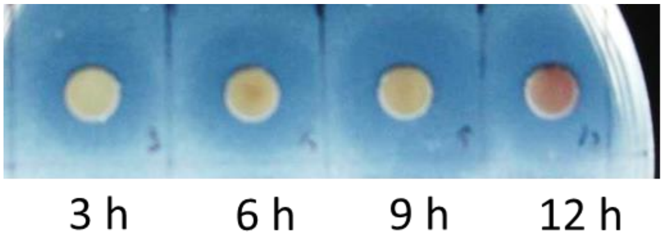

Fig. S2. The time course of extracellular keratinase production by *M. taiwanensis* WR-220. WR-220 cells were grown overnight at 55°C in 3 ml of *Thermus* modified medium. Overnight cultures were subcultured 1:100 into 100 ml fresh medium for the time course of keratinase activity analysis by harvesting 1 ml cultures per 3 hours and centrifugation at 8,000 x *g* to remove bacteria cells. Keratinase activities from the cell-free supernatants (30 μl) were measured by 1 % agarose supplemented with 1 % feather powder in 150 mM NaCl, 10 mM $CaCl_2$ and 50 mM HEPES buffer at pH8.0. After incubation overnight at 55 °C, clear zone digested by the keratinolytic protease around each disc was observed.

Figure S3.
(a)

1 MYRLVWIALL LLLASCGNRA TPDNLAPVLG LDNPNVIQGQ YIVVYKDDAN
51 VLPTLQSLKA ALDGGVTLQR ELESLGLAPD ARVEQVYTAA LLGLAARLSP
101 ENLAALRQDP RVAYIEADQV MSISATQTGA TWGLDRIDQR TLPLSGTFTY
151 SNTGSGVNAY IIDTGIRVSH SEFGGRATAV FDAIGDGQNG NDCNGHGTHV
201 AGTVGGTVYG VAKSVRLYAV RVLNCSGSGS NSGVIAGVDW VRQNARRPAV
251 ANMSLGGGAS SALDTAVNNA INAGITFALA AGNSNRDACQ FSPARVTAGI
301 TVGATTSTDA RASYSNYGSC LDLFAPGSSI TSAWISSDTS TNTISGTSMA
351 TPHVAGVAAL YLQSNPSASP ATVRNAIVGN ATSGVVSNAG RRSPNLLLYS
401 NY

(b)

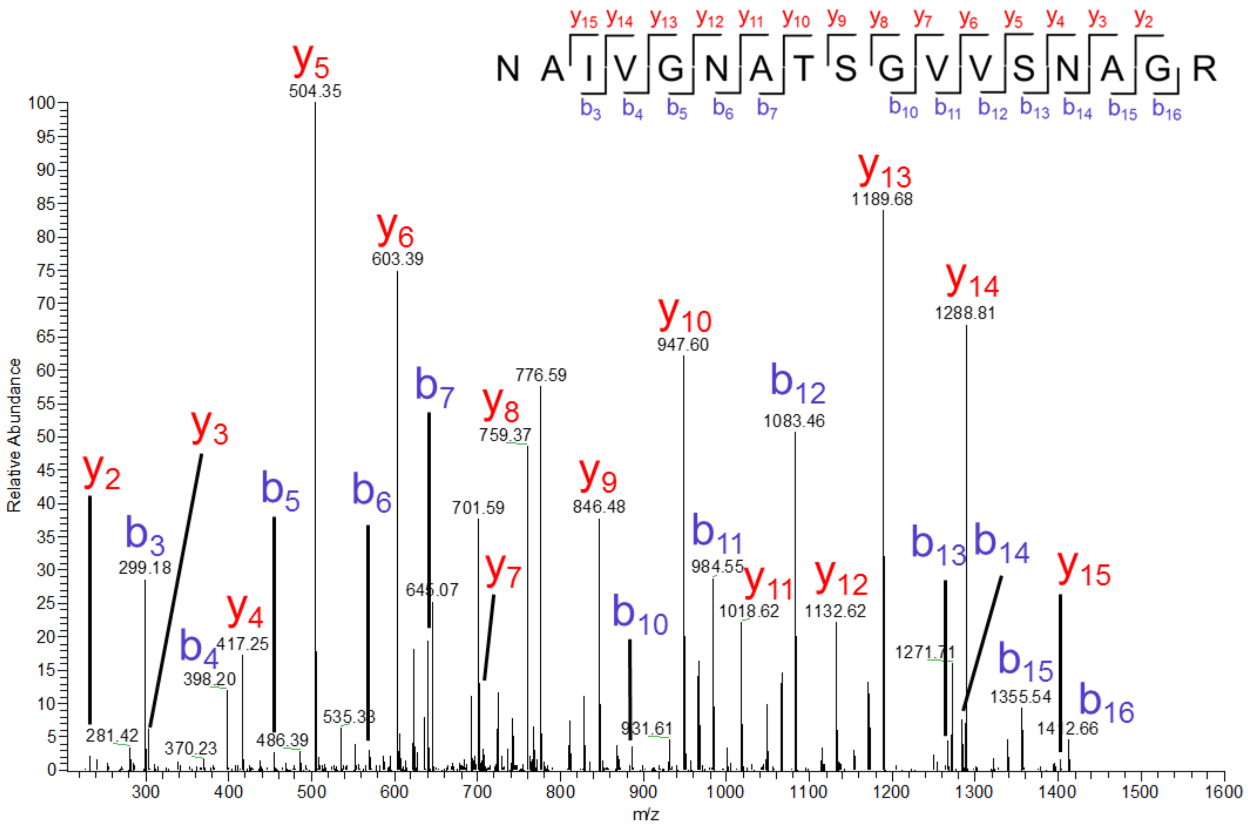

Fig. S3. Mass spectrometric identification of putative keratinase (Mtai_v1c14470).
(a) Protein sequence coverage in Mtai_v1c14470 is 27.4% and the matching peptides are shown in red. (b) MS/MS spectrum represented the identified peptide from keratinase (Mtai_v1c14470). Rich backbone fragmentation was shown in MS/MS spectrum in which the matched b and y ions were detected and shown in blue and red, respectively.

Figure S4

(a)

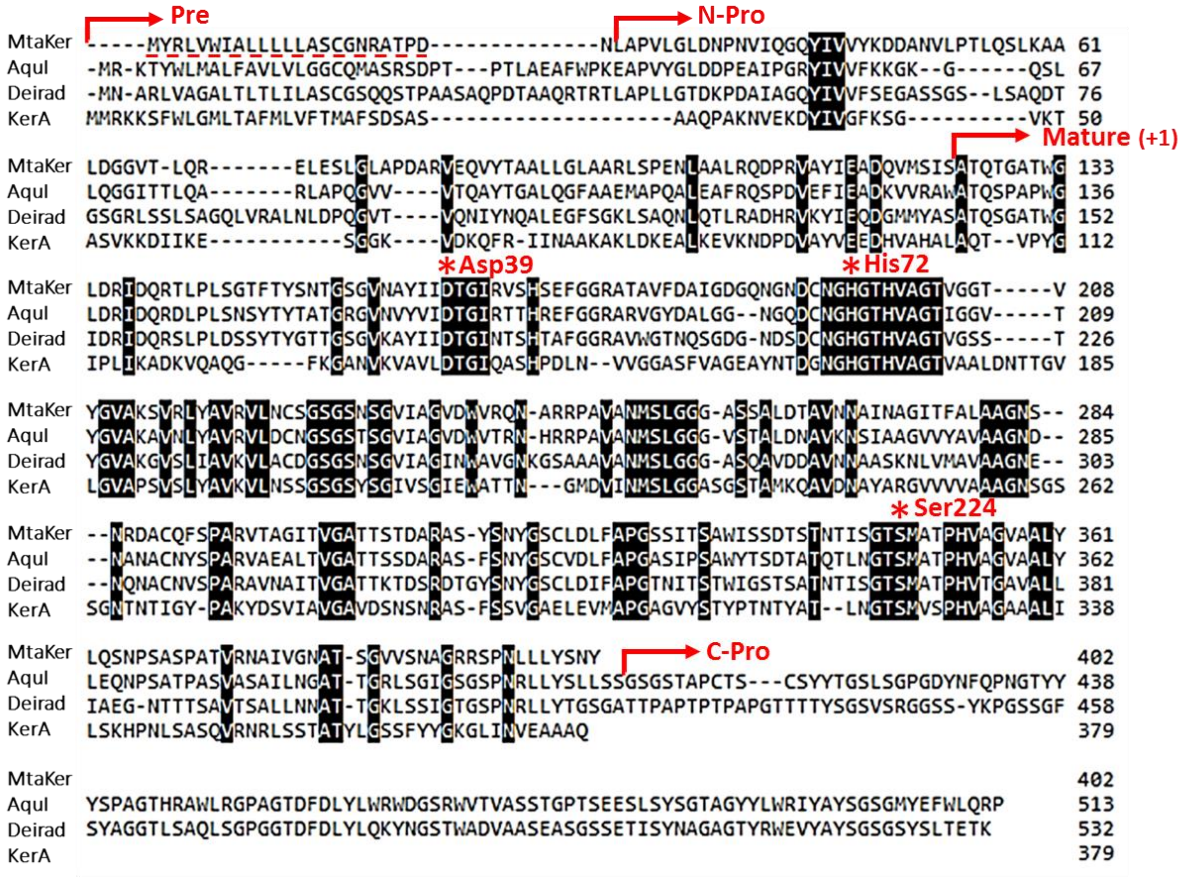

(b)

	Identity %			
	1	2	3	4
1. MtaKer	100	59.7	53.7	32.5
2. AquI		100	49.7	33.0
3. Deirad			100	28.8
4. KerA				100

Fig. S4. Amino acid sequence alignment and identity analysis of MtaKer from *M. taiwanensis* strain WR-220 with other homologous proteins and reported keratinase.

The depicted sequences are the aqualysin-1 AquI (P08594) from *Thermus aquaticus* TY1, the peptidase S8 (A0A168TGA2) from *Deinococcus radiodurans* strain R1, and the KerA (Q53521)

from *B. licheniformis* PWD-1. Red asterisks indicate the active-site residures Asp39, His72, and Ser224. Positions of the starting residues of the signal peptide (pre), N-terminal pro-peptide (N-pro), mature protease, and C-terminal pro-peptide (C-pro) are marked by red arrows. The red dashed line under MtaKer sequence indicates the signal peptide. Residues in black represent identical residues within all four proteins. (b) Comparisons of sequence identity for the multiple alignments are generated using ClustalW analysis.

Figure S5

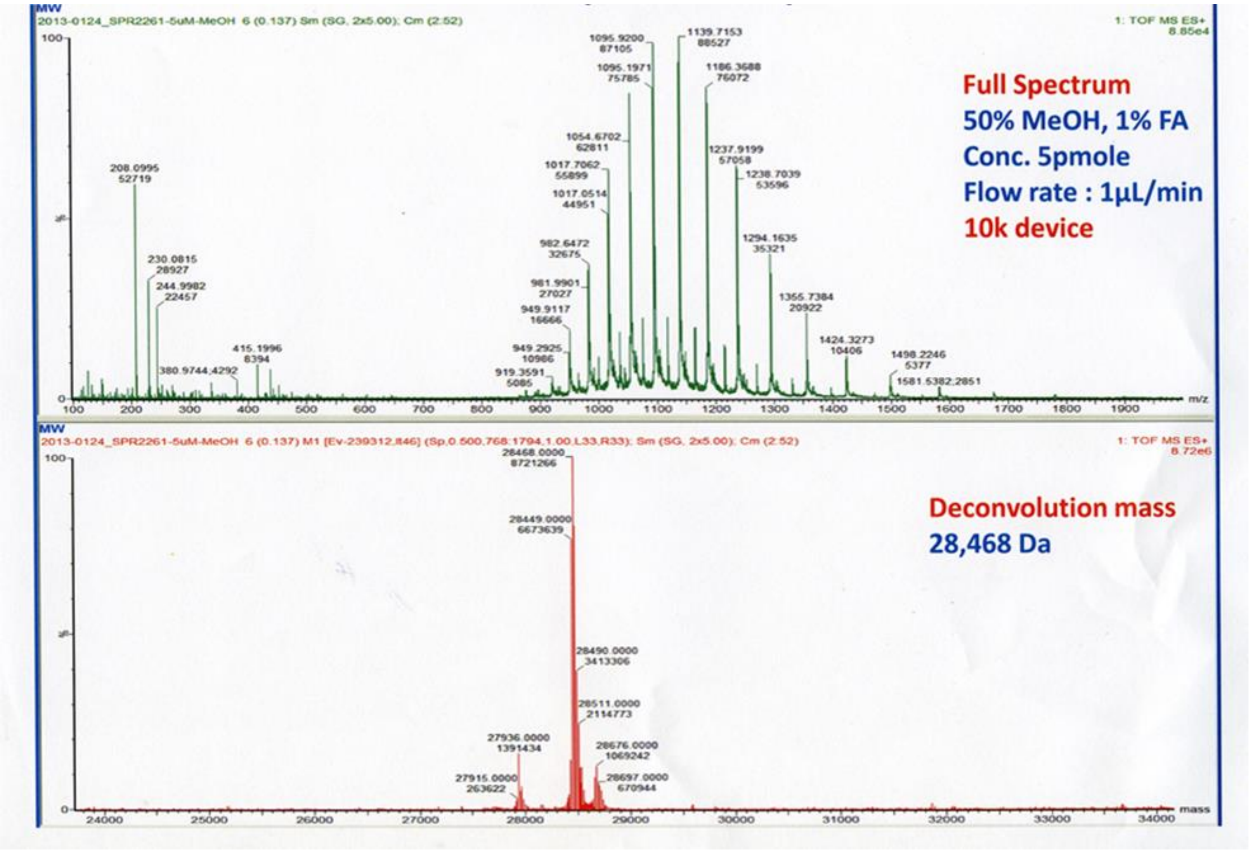

Fig. S5. The molecular weight determination of the purified MatKer analysed by LC-ESI-QTOF mass spectrometry.

The ESI mass spectrum of MatKer keratinase showed the charge state envelope after the sample was infused into the QTOF mass spectrometer. Deconvolution of the charge envelope yielded a single peak with the molecular weight determined as being 28.468 Da.

Figure S6

(a)

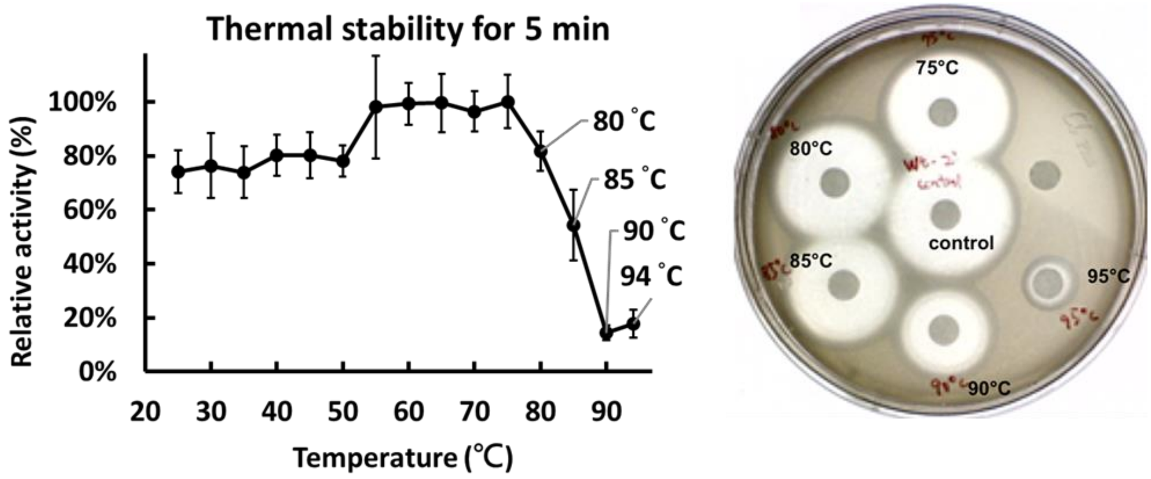

(b)

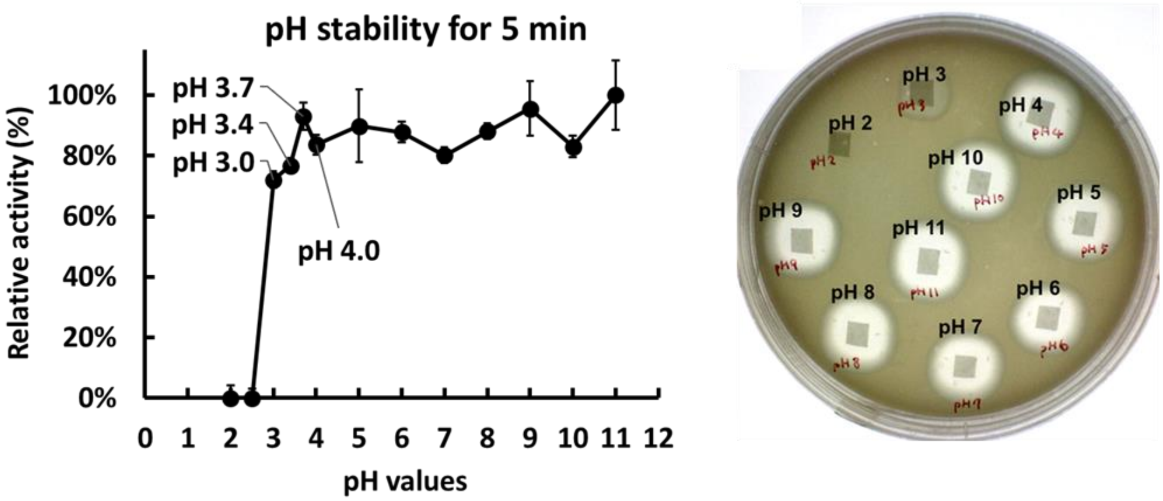

Fig. S6. The heat and pH tolerance of keratinase.

(a) The effect of rMtaKer thermostability was detected by pre-heating 5 uM of rMtaKer at different temperature values ranging from 25 to 95°C for 5 min and measured under standard condition at 55°C. The protease activity was also examined by the agar plate diffusion assay on 1 % skim milk of agar plate and shown a clear zone around the disc filter paper. The corresponding heating temperatures are indicated near the filter papers. (b) The effect of pH stability on keratinase activity was determined at 55 °C under standard condition by pre-incubating 1 uM rMtaKer in different pH solution (pH2~11) at 25°C for 5 min. The protease activity was also examined by the agar plate diffusion assay and the corresponding pH values are marketed near the rectangular filter papers.

For agar diffusion assay, The 5 mm diameter of disk filter papers (a) and squared size of filter papers (b) were impregnated with 20 μl of treated rMatKer solution and placed on milk agar plate.

Figure S7

```
       10(N-pro)   20         30         40         50         60
MLAPVLGLDN PNVIQGQYIV VYKDDANVLP TLQSLKAALD GGVTLQRELE SLGLAPDARV

        70         80         90        100 +1(mature domain) 19
EQVYTAALLG LAARLSPENL AALRQDPRVA YIEADQVMSI SATQTGATWG LDRIDQRTLP

        29         39         49         59         69         79
LSGTFTYSNT GSGVNAYIID TGIRVSHSEF GGRATAVFDA IGDGQNGNDC NGHGTHVAGT

        89         99        109        119        129        139
VGGTVYGVAK SVRLYAVRVL NCSGSGSNSG VIAGVDWVRQ NARRPAVANM SLGGGASSAL

       149        159        169        179        189        199
DTAVNNAINA GITFALAAGN SNRDACQFSP ARVTAGITVG ATTSTDARAS YSNYGSCLDL

       209        219        229        239        249        259
FAPGSSITSA WISSDTSTNT ISGTSMATPH VAGVAALYLQ SNPSASPATV RNAIVGNATS

       269        279        289        299
GVVSNAGRRS PNLLLYSNYE NLYFQSHHHH HHWSHPQFEK
```

Fig. S7. The amino acid sequence of the cloned rMtaKer from *M. taiwanensis* WR-220.

The first methionine (starting codon) and the mature form of truncated rMtaKer are labeled in bold. A fusion His-tag for protein purification are labeled in italic with lunderlined. The end part of the C-terminus (Tyr^{278}-Glu^{279}-Asn^{280}-Leu^{281}-Tyr^{282}) labeled in red was observed in the rMtaKer crystal structure as a substrate, which was buried in the active-site cavity of the neighboring monomer (in manuscript Figure 5e).

Figure S8

(a)

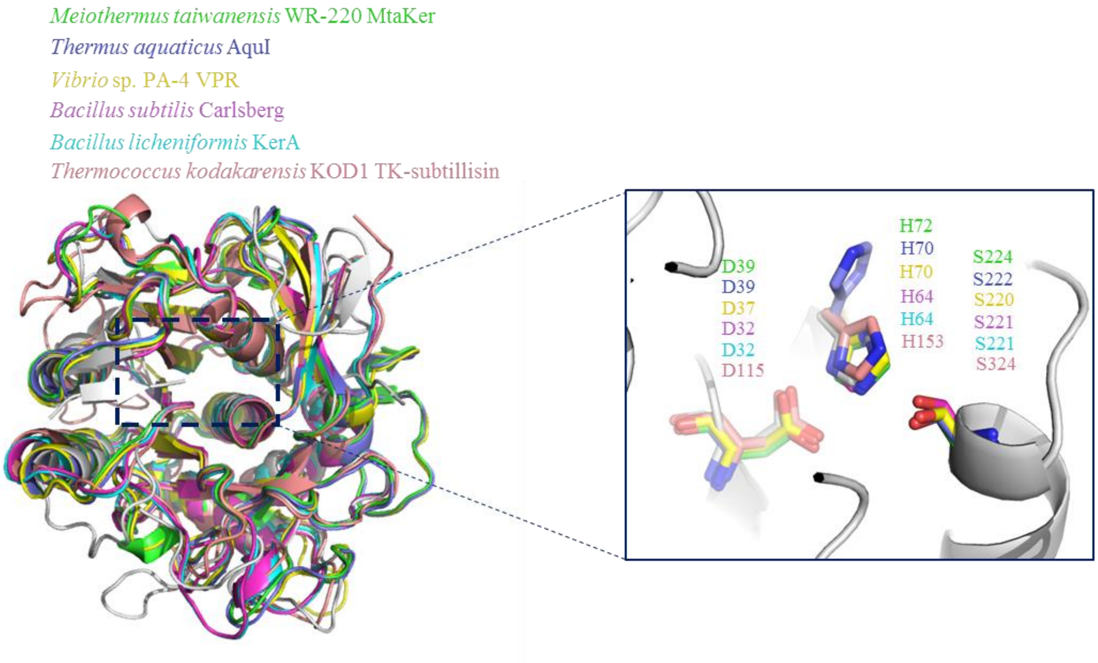

(b)

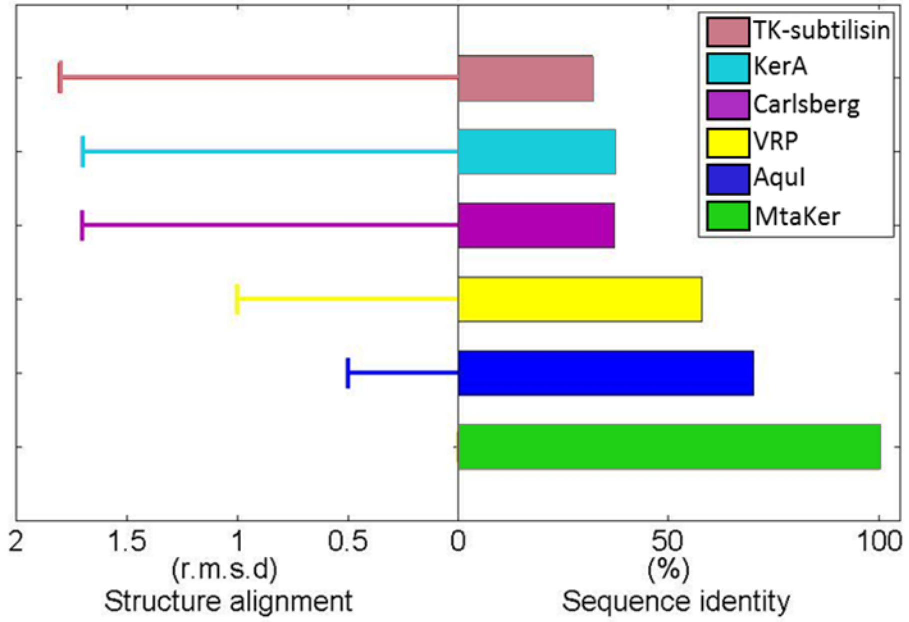

Fig.S8. Comparative structural analysis of the MtaKer mature (M) domain with other known M domain from bacterial S8 peptidases.

(a) Superimposition of the structure of the MtaKer M domain with other S8 peptidase family, including AquI (PDB ID: 4dzt), VPR (PDB ID: 1sh7), Carlsberg (PDB ID: 1cse), KerA (PDB ID:

4gi3) and Tk-subtilisin (PDB ID: 2z2x). The magnified area in the right panel is shown their overlaying catalytic residues and each of the labeled colors is corresponding to individual M domains. (b) The protein sequence and structure relationships between each M domains are shown high structural similarity (r.m.s.d < 1.8 Å) but low sequence identity from 32% to 70%. The structural similarity is determined by the low root-mean-square deviation (r.m.s.d) value. Only M domains are built the sequence alignment.

Figure S9

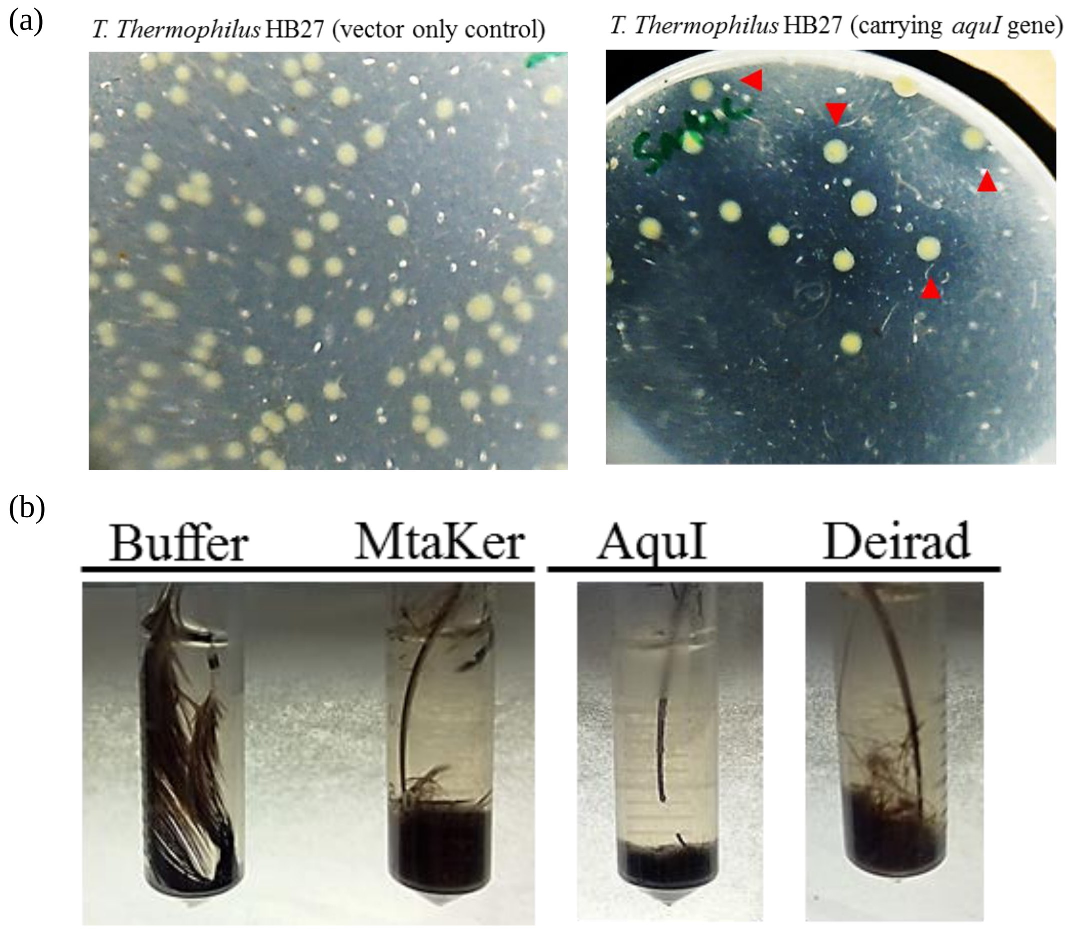

Fig. S9. Extracellular secretion of the keratinolytic aqualysin I by *Thermus thermophilus* HB27.

(a) The keratinolytic activities were determined by the appearance of a clear zone (red triangle) around single colonies when *Thermus* transformants were grown on TM agar plates containing 1% feather powder for 2 days at 70 °C. The keratinase activity of aqualysin I (*aquI*) gene was constructed in a *Thermus*-*E. coli* shuttle vector, a modified plasmid pMKE2 (Biotools B&M, Madrid, Spain) with the pilF promoter (PpilF) as described previously [2], and AquI was extracellular secreted by *T. thermophilus* HB27. (b) The purified rMtaKer and C-terminal truncated AquI and Deirad proteins were determined the keratinolytic activities using intact chicken feathers as substrate. The keratinase activity of Deirad was observed as 10 mM DTT was added to the reaction buffer.

Figure S10

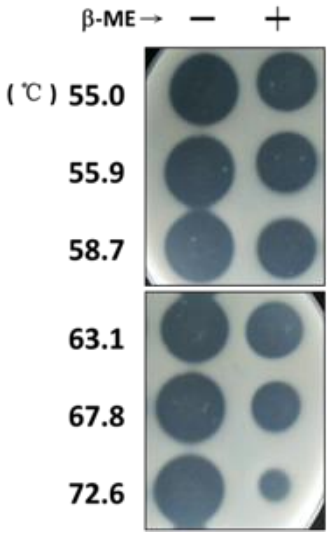

Fig. S10. The effect of reducing agent on the thermostability of rMtaKer.

5 μM rMtaKer was treated with 5 mM β-mercaptoethanol or not. After treatment, proteins were heated at the temperature ranging from 55-72.6 ^{o}C for 1 min and performed protease assay at 55 ^{o}C for 12 hours. The protease activity was determined by disk diffusion assay. The circle disk papers were impregnated with 20 μl of rMtaKer and placed on 1% agarose containing 1% skim milk. After incubation, the digested zones were visually assessed by removing the filter disk papers from agar plate.

References

1. Rosen, H. A modified ninhydrin colorimetric analysis for amino acids. *Arch Biochem Biophys* **67**, 10-15 (1957).
2. Wu, W. L. *et al.* Phosphoproteomic analysis reveals the effects of PilF phosphorylation on type IV pilus and biofilm formation in *Thermus thermophilus* HB27. *Mol Cell Proteomics* **12**, 2701-2713 (2013).
3. Cox, J. & Mann, M. MaxQuant enables high peptide identification rates, individualized p.p.b.-range mass accuracies and proteome-wide protein quantification. *Nat Biotechnol* **26**, 1367-1372 (2008).
4. Cox, J. *et al.* Andromeda: a peptide search engine integrated into the MaxQuant environment. *J Proteome Res* **10**, 1794-1805 (2011).
